# *Saccharomyces boulardii* enhances anti-inflammatory effectors and AhR activation via metabolic interactions in probiotic communities

**DOI:** 10.1093/ismejo/wrae212

**Published:** 2024-11-03

**Authors:** Karl Alex Hedin, Mohammad H Mirhakkak, Troels Holger Vaaben, Carmen Sands, Mikael Pedersen, Adam Baker, Ruben Vazquez-Uribe, Sascha Schäuble, Gianni Panagiotou, Anja Wellejus, Morten Otto Alexander Sommer

**Affiliations:** Novo Nordisk Foundation Center for Biosustainability, Technical University of Denmark, Kongens Lyngby 2800, Denmark; Department of Microbiome Dynamics, Leibniz Institute for Natural Product Research and Infection Biology—Hans Knöll Institute (Leibniz-HKI), Jena 07745, Germany; Novo Nordisk Foundation Center for Biosustainability, Technical University of Denmark, Kongens Lyngby 2800, Denmark; Novo Nordisk Foundation Center for Biosustainability, Technical University of Denmark, Kongens Lyngby 2800, Denmark; National Food Institute, Technical University of Denmark, Kongens Lyngby 2800, Denmark; Human Health Biosolution, Novonesis, Hørsholm 2970, Denmark; Novo Nordisk Foundation Center for Biosustainability, Technical University of Denmark, Kongens Lyngby 2800, Denmark; Center for Microbiology, VIB-KU Leuven, Leuven 3001, Belgium; Department of Microbiome Dynamics, Leibniz Institute for Natural Product Research and Infection Biology—Hans Knöll Institute (Leibniz-HKI), Jena 07745, Germany; Department of Microbiome Dynamics, Leibniz Institute for Natural Product Research and Infection Biology—Hans Knöll Institute (Leibniz-HKI), Jena 07745, Germany; Faculty of Biological Sciences, Institute of Microbiology, Friedrich Schiller University, Jena 07743, Germany; Jena University Hospital, Friedrich Schiller University, Jena 07743, Germany; Department of Medicine, University of Hong Kong, Hong Kong (SAR), China; Human Health Biosolution, Novonesis, Hørsholm 2970, Denmark; Novo Nordisk Foundation Center for Biosustainability, Technical University of Denmark, Kongens Lyngby 2800, Denmark

**Keywords:** *S. boulardii*, co-culture, microbial communities, probiotics, anti-inflammatory, inflammation, Genome-scale metabolic modelling, metabolic interaction

## Abstract

Metabolic exchanges between strains in gut microbial communities shape their composition and interactions with the host. This study investigates the metabolic synergy between potential probiotic bacteria and *Saccharomyces boulardii*, aiming to enhance anti-inflammatory effects within a multi-species probiotic community. By screening a collection of 85 potential probiotic bacterial strains, we identified two strains that demonstrated a synergistic relationship with *S. boulardii* in pairwise co-cultivation. Furthermore, we computationally predicted cooperative communities with symbiotic relationships between *S. boulardii* and these bacteria. Experimental validation of 28 communities highlighted the role of *S. boulardii* as a key player in microbial communities, significantly boosting the community’s cell number and production of anti-inflammatory effectors, thereby affirming its essential role in improving symbiotic dynamics. Based on our observation, one defined community significantly activated the aryl hydrocarbon receptor—a key regulator of immune response—280-fold more effectively than the community without *S. boulardii*. This study underscores the potential of microbial communities for the design of more effective probiotic formulations.

## Graphical Abstract

**Graphical Abstract:**
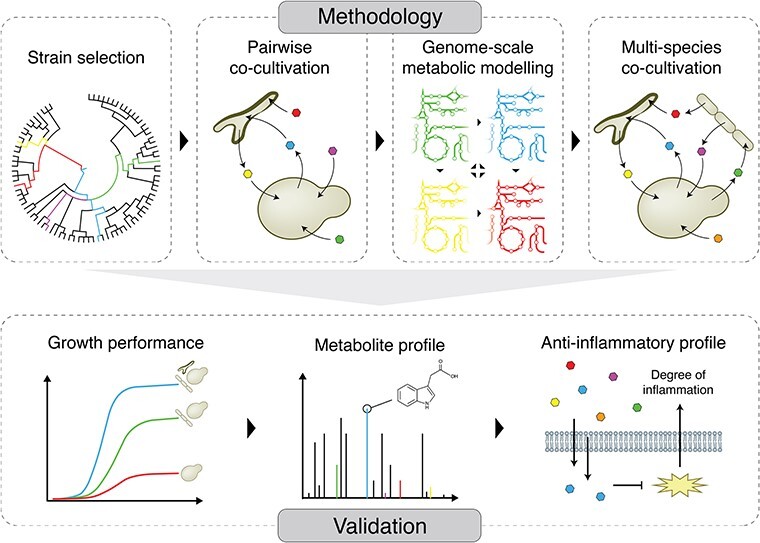

## Introduction

Microorganisms residing in complex and interactive communities are abundant in nature, thriving across diverse environmental habitats, including soil, water, plants, animals, and humans [1–3]. These microbial communities exhibit substantial diversity, ranging from small multicellular aggregates to complex communities composed of trillions of cells [4]. Among these, the human gut microbiome stands out as a highly complex and dynamic microbial ecosystem, comprising a diverse array of microorganisms [1, 5]. These communities can influence the host’s health, with various microbial species and community compositions being associated with diseases [6–8] and overall well-being [9]. Thus, altering the human gut microbiota through the supplementation of beneficial microbes has been an active research area for several years [10]. Single-strain probiotics have been reported to help manage microbial infections [11, 12], obesity [13], type 2 diabetes [13, 14], and inflammatory bowel syndrome [15] and to enhance outcomes in cancer treatment [16]. However, conflicting evidence suggests that single-strain probiotics often yield inconsistent results with minimal or no impact on human health [17, 18]. Accordingly, the therapeutic potential of probiotic microbes remains to be fully exploited, and the development of new intervention strategies is needed. A critical aspect of this exploration is to exploit the beneficial effect of microbial communities [19].

Microorganisms naturally exist within complex communities, comprising diverse species that rely on interactions to facilitate a variety of metabolic processes necessary for their survival [20, 21]. Given the longstanding history of these naturally occurring communities, there has been a growing focus on harnessing the resilience of microbial communities, including those consisting of naturally prevalent strains, for beneficial purposes [22]. Recent studies have underscored the capacity of synthetic created microbial communities to influence ecological interactions [23–25], stimulate species growth [26, 27], and facilitate the synthesis of valuable chemicals [27–29] of industrial importance. Furthermore, certain probiotic community formulations have been demonstrated to be more efficient in restoring gut ecosystem balance than a single strain [30–32]. However, a comprehensive understanding of microbial interactions is essential for developing multi-species communities [33] to mitigate the risk of antagonistic interactions [34, 35].

In natural ecosystems, a common and important phenomenon is the symbiotic relationship between yeast and lactic acid bacteria [36]. This interaction is particularly prevalent in a variety of natural food, beverage, and industrial fermentation [27, 37, 38]. Studies have demonstrated that yeast plays a vital role in supporting the growth of lactic acid bacteria by naturally providing nutrients and support through its internal cross-feeding mechanisms within established communities [36]. Furthermore, there has been a growing interest in harnessing the combined powers of yeast and lactic acid bacteria to develop potent probiotic mixtures [39]. Although such probiotic cocktails are commercially available, the clinical evidence supporting their efficacy remains limited [32, 39]. Among the various probiotics, *Saccharomyces boulardii* is the most frequently used probiotic yeast with its demonstrated efficacy in alleviating specific gastrointestinal-associated diseases, including antibiotic-associated diarrhoea, *Clostridium difficile* and *Helicobacter pylori* infections, and inflammatory bowel diseases [40].

Despite the advances in the field, particularly the interactions and mechanisms of probiotic multi-kingdom communities remain incompletely understood. Improving our comprehension and predictive capabilities could significantly enhance the therapeutic outcomes and translational potential of such communities. Central to the value of probiotics is their capacity to synthesise anti-inflammatory effectors, such as short-chain fatty acids (SCFAs) and indole-tryptophan derivatives, which have emerged as promising pathways for enhancing human health [41]. These derivatives are instrumental in promoting gut health, mitigating inflammation, and fostering the growth of commensal bacteria, thereby showcasing their wide-ranging benefits and applications. Here, we studied probiotic interactions, with a specific emphasis on the role of *S. boulardii* within microbial communities and the production of anti-inflammatory effectors. Our investigation narrowed down 85 bacterial strains to two strains with symbiotic relationships with *S. boulardii* by studying pairwise co-cultivation. Furthermore, we developed a computational approach to identify multi-species communities of three to five members with potential positive interactions, leading to the identification of one robust community with increased production of anti-inflammatory effectors.

## Materials and method

### Strains and media

All strains used in this study are listed in Supplementary Table S1. All *Bifidobacteriales* and *Lactobacillales* strains were obtained from Chr. Hansen Culture Collection. *Saccharomyces boulardii* (*Saccharomyces cerevisiae* ATCC MYA796) was obtained from American Type Culture Collection (ATCC). *S. boulardii* expressing a GFP marker was obtained as previously described [42]. All strains were either cultivated in (i) *Lactobacillus* MRS media pH 6.5 (VWR; Supplementary Table S2). The MRS media is based on the formulation by deMan, Rogosa, and Sharpe [43], with slight modifications made by the supplier company. This modified MRS media supports the luxuriant growth of all *Lactobacillus* from various sources including the oral cavity, dairy products, foods, faeces, and other sources, or (ii) modified synthetic complete media (SCmod) containing 6.7 g/l yeast nitrogen base without amino acids, 1.92 g/l Yeast Synthetic Drop-out Medium Supplement without uracil, 20 mg/l uracil, 20 g/l glucose, 5 g/l sodium-acetate, and 1 g/l Tween-80 pH 6.5 (Sigma Aldrich; Supplementary Table S2). All cultivations with *Bifidobacteriales* and the multi-species co-cultivation experiments were supplemented with 0.05% cysteine hydrochloride monohydrate (CyHCl).

### Bacterial growth assessment

Bacterial strains were cultivated overnight in 10 ml MRS media, then subcultured at 1:1000 into 200 μl of MRS in a CELLSTAR 96-well microtiter plate (Greiner Bio-One) with a TopSeal-A PLUS microplate seal (PerkinElmer). Cultivations were performed under anoxic conditions with continuous shaking at 37°C. Real-time OD_600_ was measured every 10 min for 24 h with a microplate reader (BioTek Epoch2). The anoxic condition was accomplished by growing the pre-cultures in anoxic boxes with AnaeroGen pouches (Thermo Fisher Scientific; AN0035A) and culture plates in the Coy Anaerobic Chamber (gas mixture, 95% N_2_ and 5% H_2_).

### Sterile bacterial spent media experiment

Bacterial strains were cultivated overnight in 10 ml MRS media, then subcultured 1:100 into 10 ml fresh MRS media, and cultivated for 24 h. The cultures were performed under anoxic conditions at 37°C. The anoxic condition was accomplished by growing the cultures in anoxic boxes with AnaeroGen pouches (Thermo Fisher Scientific; AN0035A). All cultures were performed in duplicates. OD_600_ was measured with a bench spectrophotometer. The cultures were centrifuged at 10000 × *g* for 10 min, and spent media was collected and filtered through a 0.22 μm vacuum filter. Spent media from duplicates were pooled together. The pH of the spent media was measured using an electronic pH meter. Spent media was neutralised with 1 M NaOH.

### Growth assessment on bacterial spent media

Pre-culture of *S. boulardii* was started from frozen cryostock and cultivated overnight in MRS before proceeding. *S. boulardii* was cultivated in 200 μl of sterile bacterial spent media in a CELLSTAR 96-well microtiter plate (Greiner Bio-One) with a TopSeal-A PLUS microplate seal (PerkinElmer). All cultures were performed under anoxic conditions with continuous shaking at 37°C with an initial OD_600_ of 0.05. Real-time OD_600_ was measured every 10 min for 24 h with a microplate reader (Agilent Technologies; BioTek Epoch2). The anoxic condition was accomplished by growing the pre-cultures in anoxic boxes with AnaeroGen pouches (Thermo Fisher Scientific; AN0035A) and culture plates in the Coy Anaerobic Chamber (gas mixture, 95% N_2_ and 5% H_2_).

### High-performance liquid chromatography

Acetic acid, butyric acid, ethanol, glucose, lactic acid, and propionic acid were all detected and quantified using high-performance liquid chromatography (HPLC). All samples were stored at >−20°C before being analysed. Dionex Ultimate 3000 HPLC system with analysis software Chromeleon (Thermo Fisher Scientific) was used for all samples. Samples were analysed with a refractive index detector and a Bio-Rad Aminex HPx87 column with 5 mM H_2_SO_4_ as an eluent at a flow rate of 0.6 ml/min with column oven temperature set to 30°C. Limit of detection for all metabolites was 0.1 g/l. The basal levels (Supplementary Table S2) of acetic acid, butyric acid, ethanol, lactic acid, and propionic acid were subtracted from each pair-wise and multi-species culture to determine the change in these metabolites.

### Quantification of tryptophan and tryptophan derivatives by LC–HRMS

Standard solutions for the four analytes, along with internal standard solutions, were prepared at a concentration of 1 mg/ml (Supplementary Table S3). The analytes were combined and diluted with 10% ethanol in MilliQ water to achieve final concentrations ranging from 0 to 200 μg/ml (0, 0.5, 1, 5, 10, 50, 100, and 200 μg/ml). An internal standard concentration of 4 μg/ml was incorporated into all standard solutions and samples. Calibration curves were then generated based on the analysis of these standard mixtures.

For each sample, a volume of 2 μl was injected into an ultra-performance liquid chromatography quadrupole time-of-flight mass spectrometry system containing a Dionex Ultimate 3000 RS liquid chromatograph (Thermo Fisher Scientific) coupled to a Bruker maXis time-of-flight mass spectrometer equipped with electrospray interphase (Bruker Daltonics) operating in negative mode. The analytes were separated on a Poroshell 120 SB-C18 column with a dimension of 2.1 × 100 mm and 2.7 μm particle size (Agilent Technologies) based on the previously suggested settings [44]. The column was kept at 40°C and the sampler at 4°C. The UPLC mobile phases consisted of 0.1% formic acid in water (solution A) and 0.1% formic acid in acetonitrile (solution B). The analytes were eluted using 1% solution B for 1 min, followed by a linear gradient up to 15% at 3 min, a linear gradient up to 50% B at 6 min, and finally a linear gradient up to 95% solution B at 9 min. This gradient was kept constant until 10 min, after which the solvent composition was returned to initial conditions at 10.1 min and re-equilibrated until 13 min. All of this occurred while the analytes were being eluted at a constant flow rate of 0.4 ml/min. Mass spectrometry data were collected in full scan mode at 2 Hz with a scan range of 50–1000 mass/charge (*m/z*). The following electrospray interphase settings were used: nebulizer pressure 2 bar, drying gas 10 l/min, 200°C, capillary voltage 4500 V. To improve the measurement accuracy, external and internal calibrations were done using sodium formate clusters (Sigma-Aldrich), and in addition a lock-mass calibration was applied (hexakis (1H,1H, 2H-perfluoroetoxy) phosphazene; Apollo Scientific).

### Flow cytometry

Pre-cultures of the strains were started from frozen cryostock and cultivated for 48 h in 2 ml of MRS media, then diluted to an OD_600_ of 0.01 with a final volume of 500 μl of either MRS or SCmod media, as specified, in a 96 deep-well plate. Cultures grown in SCmod were washed twice prior to dilution (3000*g*, 10 min, 20°C). To determine the impact of bacterial load on *S. boulardii*, six different starting OD_600_ values of bacteria (0.0001, 0.001, 0.01, 0.1, and 1.0) were tested while maintaining *S. boulardii* at an OD_600_ of 0.01. Conversely, to assess the impact of *S. boulardii* load on bacteria, six different starting OD_600_ values of *S. boulardii* (0.0001, 0.001, 0.01, 0.1, and 1.0) were tested while keeping the bacteria at an OD_600_ of 0.01. All cultures were incubated under anoxic conditions at 37°C. The anoxic condition was accomplished by growing the culture in anoxic boxes with AnaeroGen pouches (Thermo Fisher Scientific; AN0035A). For flow cytometry, 40 μl of experimental culture was taken and diluted in 160 μl filtered PBS in a clear bottom microplate at 0 h and 10 μl of experimental culture was taken and diluted in 190 μl filtered PBS in a clear bottom microplate at 24 h. Flow cytometry was performed using a Novocyte Quanteon (Agilent Technologies). FSC and SSC were measured with a gain of 400; GFP was measured using a blue laser at 525 nm and with a gain of 470; a threshold of 6000 was used. The sample was measured until 4000 events were collected in the yeast gate, or at least 30 μl of the sample was injected. Gates to identify yeast and bacteria in co-cultures were set based on events collected from mono-cultures (Supplementary Fig. S1).

### Cell culture and maintenance

The human intestinal epithelial HT-29 cell line and HT29-Lucia AhR cell line were purchased from ATCC (Catalogue number: HTB-38) and Invivogen (Catalogue number: ht2l-ahr). HT-29 cells and HT29-Lucia AhR cells were grown in McCoy’s 5A (Modified) Medium (Thermo Fisher Scientific; Gibco) supplemented with 10% foetal bovine serum (FBS) (Thermo Fisher Scientific; Gibco), and 1% penicillin–streptomycin (Thermo Fisher Scientific) in a humidified incubator at 37°C with 5% CO_2_. All analyses were conducted with three biological replicates from the co-cultures.

### LPS stimulated HT-29 cell line

HT-29 cells were thawed and passaged for three to five generations. 5000 cells in 100 μl were seeded into a CELLSTAR 96-well microtiter plate (Greiner Bio-One) and incubated for 48 h, reaching 90% confluency monolayer. Subsequently, the cells were supplemented with 100 μl of fresh McCoy’s 5A media containing 4 μg/ml LPS (Sigma Aldrich; Lipopolysaccharides from *Escherichia coli* O111:B4) and 20% spent media, resulting in final concentrations of 2 μg/ml LPS and 10% spent media. 10% (v/v) of MRS media was used in the control groups. Spent media was generated by cultivating the bacteria and *S. boulardii* in MRS media for 24 h. The cultures were centrifuged at 10000 × *g* for 10 min, and spent media was collected and filtered through a 0.22 μm syringe filter. Supernatants from the treated cell lines were collected by spinning down the culture after 24 h. IL-8 concentration in the culture supernatant was measured using Human IL-8 ELISA (Abcam; ab214030), according to the manufacturer’s instructions. Viability was assessed by measuring lactate dehydrogenase levels in the supernatant using the CyQUANT LDH Cytotoxicity Assay kit (Thermo Fisher Scientific; Catalogue number: C20300), according to the manufacturer’s instructions.

### Aryl hydrocarbon receptor cell line

HT29-Lucia AhR cells were thawed and passaged for two generations in the absence of the selection antibiotic Zeocin (Invivogen; CAS number: 11006-33-0). Subsequent passages were maintained in 100 μg/ml Zeocin and 1% penicillin–streptomycin. Cells were harvested before reaching 90% confluency. 5000 cells per well were seeded into 96-well plates (Greiner Bio-One; Catalogue number: 655160) in 160 μl volume. 40 μl of spent media, MRS media as negative control, or 20 μM FICZ (Sigma-Aldrich, CAS number: 172922-91-7) as a positive control, was added into the well and incubated for 48 h. Spent media was generated by cultivating the bacteria and *S. boulardii* in MRS media for 24 h. The cultures were centrifuged at 10000 × *g* for 10 min, and spent media was collected and filtered through a 0.22 μm syringe filter. Following incubation, 20 μl of supernatant was transferred to a black 96-well plate with optical bottom (Thermo Fisher Scientific; Catalogue number: 165305). 50 μl QUANTI-Luc: Luciferase Detection Reagent (Invivogen; Catalogue number: rep-qlc4r2) was added to each well, and luminescence was immediately read on a BioTek Synergy H1 plate reader (Agilent Technologies) using 2 mm read-height, 200 ms integration time, and 100 gain.

### Metabolic modelling

The Yeast consensus genome-scale metabolic model v8.6.2 was downloaded from https://github.com/SysBioChalmers/yeast-GEM/releases [45]. 13 genome-scale metabolic models for the studied bacterial species were downloaded from the CarveMe repository (Table 1) [46]. The Lactobacillus_johnsonii_DPC_6026 genome-scale metabolic model was downloaded from AGORA v1.03 collection of genome-scale metabolic models (Table 1) [47]. 50 bacterial genome-scale models were randomly selected (Supplementary Table S4) and downloaded from https://github.com/cdanielmachado/embl_gems/tree/master. In addition, three fungal genome-scale models of *Penicillium chrysogenum* (iAL1006) [48], *Aspergillus niger* (iMA871) [49], and *Aspergillus oryzae* (iWV1314) [50] were used as random fungal models for test purposes. iAL1006 was downloaded from the supporting information [48]. iMA871 and iWV1314 were downloaded from the BioModels repository (https://www.ebi.ac.uk/biomodels/) with the Model IDs MODEL1507180047 and MODEL1507180056, respectively. The IDs of metabolites of the Yeast, random fungal models, and *Lactobacillus johnsonii* genome-scale metabolic models associated with exchange were manually mapped to the BiGG [51] nomenclature, which is already present for the CarveMe models. All the used models are available at https://github.com/mohammadmirhakkak/S_boulardii_bacterial_communities/tree/main/GEMs. Gene-to-reaction annotations for metabolic reactions that convert boundary metabolites, that is, metabolites for which exchange reactions exist, are summarised in Supplementary Table S5. All possible combinations of microbial communities with three and four bacterial genome-scale metabolic models with and without the Yeast genome-scale metabolic model were generated and used as input for SMETANA analysis [52]. SMETANA (v1.2.0) was used with the default settings except for the attribute—flavour (set to “ucsd”). The attribute—mediadb was used to add details of SCmod media composition (media components as presented in Supplementary Table S6). MRS media was simulated by not setting the—mediadb attribute, which simulates complete media access as given by exchange reactions in any model under investigation. SMETANA was run in two modes (“general” and “detailed”) to provide the assessments for cooperation (metabolic interaction potential or MIP), competition (metabolic resource overlap or MRO), and potential metabolite exchanges (SMETANA score) within each community. In brief, MIP represents the difference between the minimal number of components required for the growth of all members in a noninteracting community compared to an interacting community. Here, member species are exclusively using nutrients in a noninteracting community, whereas species in an interacting community are allowed to use both nutrients and secreted metabolites by other community members. In contrast, MRO estimates the theoretical possible overlap between the minimal nutritional requirements of all member species, as is provided by MIP scores. The SMETANA score for a community indicates the growth dependency of species A on metabolite m produced by species B which is calculated as a product of three separate scores: (i) species coupling score (SCS), (ii) metabolite uptake score (MUS), and (iii) metabolite production score (MPS). (i) The SCS measures the dependency of the growth of a given species A on the presence of another species B in a community of N members. (ii) The MUS measures the growth dependency of a given species A on metabolite m donated by the other community members. (iii) MPS employs a linear programming (LP) problem to calculate a binary score indicating whether a given species B can produce metabolite m (MPS = 1) or not (MPS = 0) in the community of N members. Further details on including the (M)ILP formulation can be found in the original publication and its supporting information [52] as well as online at https://smetana.readthedocs.io.

**Table 1:** List of bacterial genome-scale metabolic models.

No.	Alias	Bacterial genome-scale metabolic model	Source
1	*B. longum*	B_longum_subsp_infantis	1
2	*L. acidophilus*	L_acidophilus	1
3	*L. delbrueckii*	L_delbrueckii_subsp_delbrueckii	1
4	*L. gasseri*	L_gasseri	1
5	*L. paracasei*	L_paracasei	1
6	*L. salivarius*	L_salivarius	1
7	*L. brevis*	Lactobacillus_brevis_ATCC_367	2
8	*L. buchneri*	Lactobacillus_buchneri_CD034	2
9	*L. crispatus*	Lactobacillus_crispatus_125_2_CHN	2
10	*L. jensenii*	Lactobacillus_jensenii_SNUV360	2
11	*L. johnsonii*	Lactobacillus_johnsonii_DPC_6026	3
12	*L. reuteri*	Lactobacillus_reuteri_DSM_20016	2
13	*L. rhamnosus*	Lactobacillus_rhamnosus_GG_GG_ATCC_53103	2

Source: 1. https://github.com/cdanielmachado/carveme_paper/tree/master/models/gut_models/CarveMe [46]. 2. https://github.com/cdanielmachado/embl_gems/tree/master/models [46]. 3. https://vmh.life/#microbe/Lactobacillus_johnsonii_DPC_6026

To guarantee that all models can simulate growth on SCmod media (reflected by non-zero biomass values per model), we systematically investigated individual model capabilities for metabolite uptakes. Briefly, in addition to SCmod media components we relaxed all other exchange flux bounds and tracked growth prediction by iteratively removing additional exchange fluxes again. If any compound was found to be essential for growth it was added to the SCmod media list, and the remainder of additional exchange fluxes were again investigated for essentiality with regards to growth until we identified a minimal set of additionally required compounds for model simulation. We identified 78 essential additional compounds in total over all models. Consequently, these were added to the simulated SCmod media to prevent bias in any individual model simulation. The simulated composition of the SCmod can be found in Supplementary Table S6.

All analyses including flux balance and flux variability analysis were done in Python (v3.6) with IBM CPLEX (v12.8) solver (academic license) and COBRApy (v0.17.1) [53]. All codes and data to reproduce our *in silico* simulations are available at github: https://github.com/mohammadmirhakkak/S_boulardii_bacterial_communities.

### Statistical testing

Statistical analyses were conducted using RStudio version 4.1.0, utilising the rstatix and DescTools packages. Data are presented as mean ± SEM unless otherwise specified. A significance threshold was established at *P* < .05. For comparisons between two groups, either a dependent sample *t*-test or paired Wilcoxon-signed rank test was employed. In cases of multiple comparisons, either false discovery rate adjustments were applied, or One-way ANOVA with Tukey HSD adjustment. The phylogenetic tree was constructed using phyloT v2 (https://phylot.biobyte.de/) [54] based on NCBI Taxonomy to generate a Newick file. The Newick file was loaded in RStudio, and the tree was built using the ape and ggtree packages.

## Results

### Growth dynamics of *S. boulardii* in bacterial spent media reveal positive and negative growth interactions.

**Figure 1:**
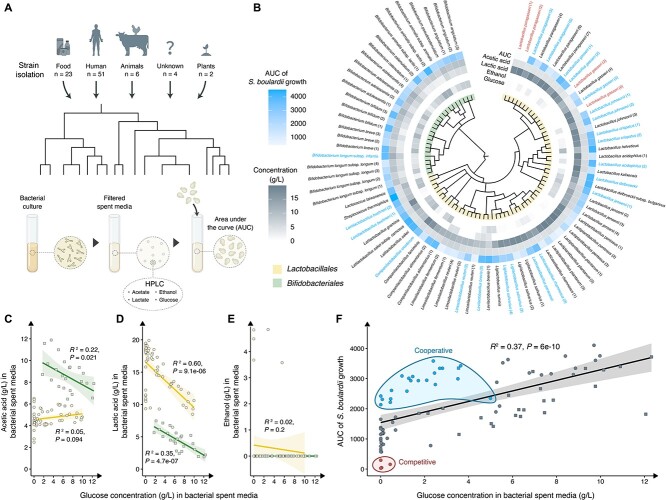
Screening of *S. boulardii* growth performance on bacterial spent media. (A) Graphical illustration of the workflow of screening *S. boulardii’s* growth performance on spent media from 85 bacterial strains. (B) *S. boulardii’s* growth performance presented as the area under the curve (AUC) and absolute end concentration of acetic acid, lactic acid, ethanol, and glucose (g/l) in neutralised bacteria spent media. Phylogenetic tree was generated by phyloT v2 based on NCBI Taxonomy, where cooperative (blue) and competitive (red) strains are highlighted. (C) Acetic acid concentration (g/l), (D) lactic acid concentration (g/l), and (E) ethanol concentration (g/l) in neutralised bacterial spent media correlated with glucose concentration (g/l) in the neutralised bacterial spent media. Circle yellow data points show *Lactobacillales* and square green data points show *Bifidobacteriales*. The dark yellow/green line indicates the best fit as determined by least square linear regression analysis with Pearson correlation; the yellow/green shaded area indicates the 95% confidence interval. (F) *S. boulardii* growth performance correlated with glucose concentration of neutralised bacterial spent media. Each data point represents the mean AUC of three replicates. Cooperative candidates (defined as >2100 AUC and <5 g/l glucose) are clustered and coloured blue. Competitive candidates (defined as <300 AUC) are clustered and coloured red. Remaining grey data points are candidates that were not selected for further investigation. Circle data points show *Lactobacillales* and square data points show *Bifidobacteriales*. The black line indicates the best fit as determined by least square linear regression analysis with Pearson correlation; the grey shaded area indicates the 95% confidence interval. Significance level at *P* < .05.

To explore the dynamic interaction between probiotic bacteria and *S. boulardii*, we assembled a diverse collection of potential probiotic bacteria (Supplementary Table S1). Three criteria were considered when selecting the strains: (i) clinical relevance; (ii) isolation sources; and (iii) phylogenetic diversity of the species. Based on these criteria, we selected 85 bacterial strains including members representing the two most prevalent probiotic classes: *Bifidobacteriales* and *Lactobacillales* [55]. These strains were sourced from various origins, including different foods, plants, animals, and the human body (Fig. 1A, Supplementary Table S1). The naming of the *Lactobacillales* follows the 2020 reclassification guidelines [56]. The numbers in parentheses indicate the specific strain numbers. Our initial step involved assessing the growth of each strain under anoxic conditions in MRS media over 24 h. The results revealed that 53 of the selected bacterial strains exhibited significantly faster growth rate than *S. boulardii* (Supplementary Fig. S2), and only six bacterial strains displayed slower growth compared to *S. boulardii*.

To study the interaction between bacteria and *S. boulardii* while ensuring that no single population dominated the culture, we cultivated *S. boulardii* in bacterial spent media obtained from the 85 bacteria strains (Fig. 1A). We observed a strong correlation between *S. boulardii* growth and the pH of the bacterial spent media (Supplementary Fig. S3). As a result, we adjusted the pH of the spent media to 6.5 to minimise any unintended impact on *S. boulardii* growth [57]. Following spent media neutralisation, we observed an enhancement in the growth performance of *S. boulardii* (Fig. 1B). To pinpoint potential metabolites exerting significance on *S. boulardii* growth, we quantified a panel of organic acids and glucose within the bacterial spent media (Fig. 1B). The acetic acid concentration in the neutralised spent media of *Bifidobacteriales* showed a significant negative correlation with glucose concentration (Fig. 1C), indicating that the *Bifidobacteriales* convert glucose to acetic acid. Additionally, the lactic acid concentration in the neutralised spent media of both bacterial phyla demonstrated a negative correlation with glucose concentration (Fig. 1D), indicating that they also convert glucose to lactic acid. Neither phylum demonstrated any correlation between ethanol and glucose concentration (Fig. 1E). Among these metabolites, unutilised glucose unsurprisingly emerged as a key compound with a pronounced impact on *S. boulardii* growth (Fig. 1F). However, also higher levels of acetic acid per utilised glucose showed to correlate with better growth performance of *S. boulardii* (Supplementary Fig. S4). Other organic acids demonstrated no significant difference (Supplementary Fig. S4).

Subsequently, we were able to identify 22 neutralised spent media in which most of the glucose had been utilised (glucose concentrations below 5 g/l), that still displayed a robust growth performance of *S. boulardii* (AUC > 2100; Fig. 1F). Furthermore, our screen also uncovered four spent media that limited the growth of *S. boulardii*, which we have classified as competitive strains.

### Pairwise co-cultivation of *S. boulardii* and probiotic bacteria is highly dependent on media conditions.

Based on the results of the neutralised spent media screening, we proceeded to investigate pairwise cultivation of the bacteria and *S. boulardii*. We selected the 22 bacterial candidates from the spent media screening, along with the four bacteria candidates that exhibited inhibitory effects on *S. boulardii* growth. It is well-established that nutrient availability in a given media influences the growth of microbial communities [58]. Therefore, we carried out pairwise co-cultivations in two distinct media, each optimised for either the bacteria or *S. boulardii*. The two media were (i) MRS media, a nutrient-enriched medium designed to support the growth of *Lactobacillales* species [43], and (ii) SCmod media, a modified synthetic complete medium tailored for *Saccharomyces cerevisiae* (Supplementary Table S2). This approach allowed us to investigate two unique scenarios where the media favour one organism over the other.

**Figure 2:**
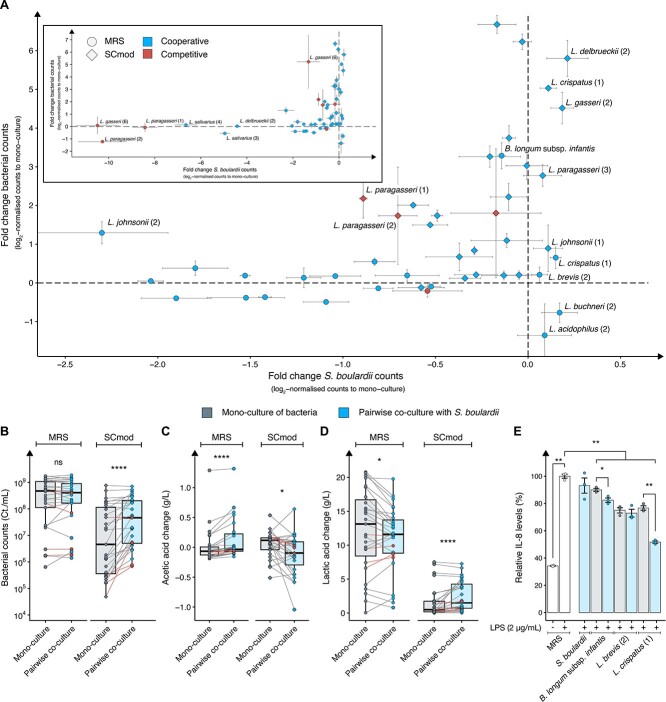
Pairwise co-culture of *S. boulardii* and 26 bacterial strains. (A) Scatter plot for visualising bacteria and *S. boulardii* enrichment in pairwise co-culture. Shown in the *y*-axis: log_2_ fold change of bacterial counts in pairwise co-culture over the bacterial counts in mono-culture and shown on the *x*-axis: log_2_ fold change of *S. boulardii* counts in pairwise co-culture over the *S. boulardii* mono-culture. Each pairwise co-culture is normalised to its respective mono-culture media conditions. The top left part of the scatter plot provides a zoomed-out overview covering the entire dataset (*x*-axis: −11:1). The main portion of the scatter plot offers a zoomed-in view (*x*-axis: −2.5:1), allowing for a closer examination of the differences and finer details within that specific area. Blue dots represent pairwise co-culture of bacterial strains predicted to have good performance with *S. boulardii* based on initial spent media screening, whereas red dots represent pairwise co-culture of bacteria predicted to have poor performance. Circle shapes indicate pairwise co-cultures in MRS media and diamond shape indicates pairwise co-cultures in SCmod media. Data are presented as the mean of three replicates ±SD. End point values of (B) bacterial counts, and changes in (C) acetic acid concentration, and (D) lactic acid concentration between mono-culture and pairwise co-culture in MRS and SCmod media. Basal level of acetic acid (MRS = 5.19, SCmod = 5.11) and lactic acid concentration (MRS = 0.16, SCmod = 0) were subtracted from each sample to determine the change in concentration. Each data point represents the mean of three replicates from either a mono-culture or a co-culture. Red lines represent the pairwise comparison of the bacteria predicted to have poor performance. (E) Relative IL-8 production in the HT-29 cell line challenged with LPS (2 μg/ml) and either 10% (v/v) MRS media (control; white) or 10% (v/v) spent media with (blue) and without (grey) *S. boulardii* for 24 h. Data are presented as the mean of three replicates ±SEM. *P* values were computed for panels B, C, and D using Wilcoxon signed rank test; and for panel E using independent two sample *t*-test and adjusted for multiple comparison with false discovery rate. Significance level at *P* < .05, ^*^P < .05, ^*^^*^P < .01, ^*^^*^^*^P < .001, and ^*^^*^^*^^*^P < .0001.

To distinguish between bacteria and *S. boulardii* populations in the communities, we employed flow cytometry (Supplement Fig. S1; Methods). The presence of *S. boulardii* showed a minimal impact on the general bacterial population in MRS media, whereas in SCmod media, it led to a 5-fold increase in bacterial counts (Fig. 2A and B). Conversely, *S. boulardii* was significantly negatively affected by most of the bacteria in MRS media (Supplementary Fig. S6). In contrast, in SCmod media, *S. boulardii* was in general less impacted by the bacteria’s presence, with only three bacterial strains significantly reducing the cell counts. Pairwise co-culture of *S. boulardii* with *L. brevis* (2) and *L. crispatus* (1) were the only co-cultures in MRS media where both the bacteria and *S. boulardii* showed tendency in improved counts compare to its respective mono-culture (Fig. 2A). *L. crispatus* (1) was the only strain performing in the top-right corner in both MRS and SCmod media. The initial ratio between the bacteria and *S. boulardii* significantly influences the end outcome of the cultures (Supplementary Fig. S5). Increasing the starting OD_600_ of bacteria negatively correlates with *S. boulardii* ability to grow. However, the effect of increasing the starting OD_600_ of *S. boulardii* is strain-dependent: *L. brevis* (2) shows a negative correlation, whereas *L. crispatus* (1) demonstrates a positive correlation.

Among the four bacterial candidates, which exhibited an inhibitory effect on *S. boulardii* growth from the neutralised spent media experiment, three of them demonstrated a significant decrease in *S. boulardii* cell numbers in both MRS and SCmod media compared to the *S. boulardii* mono-culture. *Lactobacillus gasseri* (6) exhibited the most pronounced negative effect, leading to a remarkable reduction in *S. boulardii* cell numbers by a factor of 10.3-fold (log_2_) in MRS and 1.3-fold (log_2_) in SCmod media (Fig. 2A).

Previous research has established lactic acid and SCFAs as substantial contributors to the probiotic efficacies observed by *Bifidobacteriales*, *Lactobacillales*, and *S. boulardii* [39, 59, 60]. Hence, we sought to analyse the composition in the spent media of both mono-cultures and pairwise co-cultures, aiming to assess the metabolic changes of lactic acid, SCFAs, ethanol, and glucose utilisation in the cultures with and without *S. boulardii*. During the co-cultivation of bacteria and *S. boulardii*, we observed significant alterations in the levels of acetic acid (Fig. 2C) and lactic acid (Fig. 2D) in the pairwise co-culture compared to their respective mono-cultures. In the MRS media, we observed a significant increase in acetic acid production and a simultaneous reduction in lactic acid production (Fig. 2C; Supplementary Fig. S6). Conversely, in SCmod media, we observed a significant increase in acetic acid consumption along with an elevation in lactic acid production (Fig. 2D; Supplementary Fig. S6). The quantified metabolic profile for the four bacterial candidates with competitive interaction did not exhibit any discernible differences when compared to the candidates expected to have cooperative interaction (Supplementary Fig. S6). Only one strain, *C. crustorum*, showed detectable levels of propionic acid (Supplementary Fig. S7). Moreover, pairwise co-cultivation of *C. crustorum* and *S. boulardii* resulted in 2-fold higher propionic acid levels in MRS media.

To evaluate whether these changes in metabolites affect the probiotic efficacy of mono- and pairwise co-cultivation, we assessed their anti-inflammatory properties under controlled conditions. The secretion of SCFAs and other metabolites by *Bifidobacteriales*, *Lactobacillales*, and *S. boulardii* is well-documented to play a crucial role in their probiotic efficacy through interaction with epithelial cells and promotion of anti-inflammatory responses [40, 59, 60]. Therefore, we analysed whether pairwise co-cultivation of the two top-performing strains in MRS media, *L. brevis* (2) and *L. crispatus* (1), along with *Bifidobacterium longum* subsp. *infantis*—which demonstrated a remarkable 3-fold (log_2_) increase in bacterial counts when co-cultivated with *S. boulardii*—affected the anti-inflammatory properties of specific metabolites produced by the individual strains. To assess this, we examined the modulation of interleukin-8 (IL-8) secretion in human intestinal epithelial cells (HT-29) upon stimulation with 2 μg/ml lipopolysaccharide (LPS, endotoxin). LPS induces inflammation by binding to Toll-like receptor 4 (TLR4) on the surface of HT-29 cells [61]. This binding triggers a signalling pathway that results in the production and secretion of pro-inflammatory cytokines, including IL-8. A reduction in IL-8 levels indicates a decrease in the inflammatory response, suggesting that the treatment has an anti-inflammatory effect. Spent media was harvested from mono-culture and pairwise co-cultivation in MRS media, as this medium resulted in higher overall bacterial counts and higher acetic acid production. Treatment with spent media from mono-culture of *B. longum* subsp. *infantis, L. brevis* (2), and *L. crispatus* (1) demonstrated a significant reduction in IL-8 secretion (Fig. 2E). Pairwise co-cultivation of *B. longum* subsp. *infantis* and *L. crispatus* (1) with *S. boulardii* further improved the anti-inflammatory response compared to respective mono-culture. Further testing of pairwise co-cultivation involving *Lactobacillus acidophilus* (2), *Lactobacillus buchneri* (2), and *L. paragasseri* (2) with *S. boulardii*, each of which exhibited the most negative impact on their respective bacterial counts, revealed a reverse tendency in terms of relative IL-8 levels compared to their mono-cultures (Supplementary Fig. S8), indicating that the growth performance of each bacteria plays a role in reducing IL-8.

### Genome-scale metabolic modelling predicts yeast to stabilise larger microbial communities.

Following our pairwise co-cultivation experiments, we successfully identified two communities, consisting of *L. brevis* (2) and *L. crispatus* (1), which demonstrated a tendency towards beneficial interaction with *S. boulardii*. Next, we wanted to test *S. boulardii* synergy with larger complex communities consisting of multiple bacterial species. Experimentally testing all possible three, four, or five strain combinations would result in thousands of different combinations which was beyond the scope of this study. To streamline this process, we employed genome-scale metabolic modelling to identify communities that could interact positively by applying species metabolic interaction analysis (SMETANA; Methods) [52].

**Figure 3:**
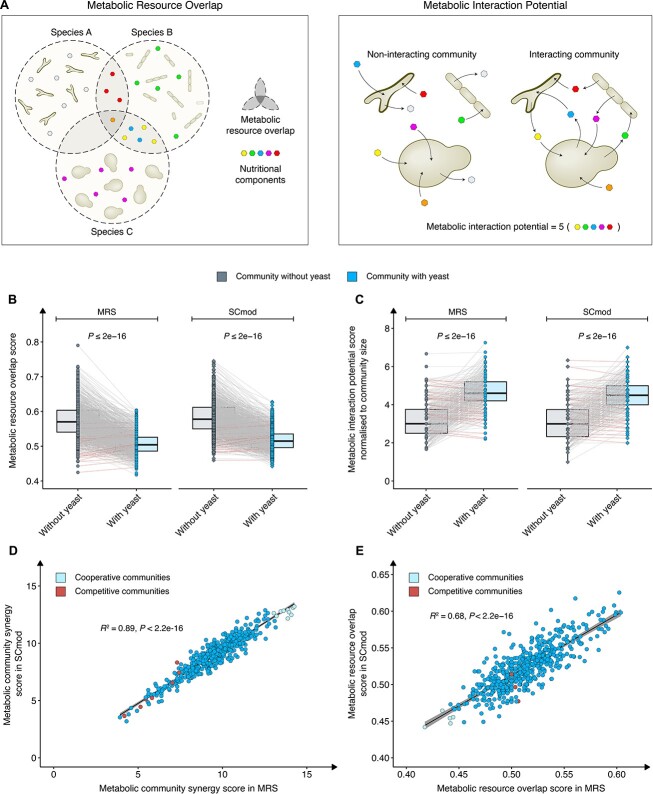
Computational assessment of strain metabolic interactions with and without yeast. (A) A graphical illustration of the concept of metabolic resource overlap, defined as the maximum possible overlap between the minimal nutritional requirements of all species within the community. Additionally, a graphical illustration of the concept of metabolic interaction potential is defined as the maximum number of essential nutritional components that a community can self-sustain through interspecies metabolic exchange. *In silico* computed (B) metabolic resource overlap score and (C) metabolic interaction potential score for different communities with and without yeast under rich undefined media (MRS simulated media) and minimal defined media (SCmod). (D) *In silico* computed metabolic community synergy score (defined as the ratio of metabolic interaction potential to metabolic resource overlap, normalised by the community size) in MRS simulated media plotted against SCmod media. (E) *In silico* computed metabolic resource overlap score (for communities lacking metabolic interaction potential score) in MRS simulated media plotted against SCmod media. The black line indicates the best fit as determined by least square linear regression analysis with Pearson correlation, and the grey shaded area indicates the 95% confidence interval. Light blue (cooperative) and red (competitive) points indicate candidates selected for further validation. The competitive communities were selected based on the analysis presented in Supplementary Fig. S10. *P* values were computed using the Wilcoxon signed rank test. Significance level at *P* < .05.

Previous research has demonstrated that resource competition tends to drive interactions in communities [52], thereby influencing community composition through competition for metabolic resources. To assess the degree of metabolic competition among potential communities, we quantified their metabolic resource overlap [52]. Metabolic resource overlap (MRO) is defined as the maximum possible overlap between the minimal nutritional requirements of all species within the community (Fig. 3A; Methods). Additionally, another crucial factor for a successful community is to display an efficient exchange of metabolites. To evaluate the tendency of these communities to exchange metabolites, we assessed their metabolic interaction potential (MIP) [52]. Metabolic interaction potential is defined as the maximum number of essential nutritional components that a community can provide for itself through interspecies metabolic exchange (Fig. 3A; Methods). A higher metabolic interaction potential score indicates a greater potential for the community to benefit from the complementary biosynthetic capabilities of its member species. In our simulated communities, a consistent pattern emerged with a reduction in metabolic resource overlap (Fig. 3B) and a corresponding increase in metabolic interaction potential (Fig. 3C) when yeast is present in the community, independent of the specific media used. To investigate whether this is a consistent general effect by adding the yeast model to a bacterial community, we generated 20× equally sized random sets with three and four bacterial models (Supplementary Table S4) and one additional fungal model randomly picked from *Penicillium chrysogenum* (iAL1006) [48], *Aspergillus niger* (iMA871) [49], or *Aspergillus oryzae* (iWV1314) [50]. Comparing the changes in metabolic resource overlap and metabolic interaction potential scores of our communities, we observed a significant overall improvement in cooperation for metabolites upon adding the yeast model compared to adding a random fungal model (Supplementary Fig. S9, Supplementary Table S4).

From our simulated communities with yeast, only a few demonstrated an elevated metabolic resource overlap score and reduced metabolic interaction potential score in the presence of yeast (Fig. 3B and C, Supplementary Fig. S10). Upon examining the performance of individual species within the community, we were able to pinpoint seven species that exerted a more substantial influence on the interaction score. Although yeast had the most significant impact on the communities, a community comprising of *B. longum*, *L. gasseri*, *Lactobacillus jensenii*, or *Lactobacillus reuteri* performed better than others in our modelling (Supplementary Fig. S11). Contrarily, *Lactobacillus salivarius* and *Lactobacillus rhamnosus* were associated with communities exhibiting lower scores.

To identify communities with potential synergy, we calculated the metabolic community synergy (MCS), defined as the ratio of metabolic interaction potential to metabolic resource overlap, normalised by community size. This allowed us to identify the top 10 communities with the highest metabolic community synergy scores in both MRS and SCmod media (Fig. 3D). A strong correlation in metabolic community synergy scores between the two different media types was observed. Although a metabolic community synergy score provides a comprehensive summary of metabolic resource overlap, metabolic interaction potential, and community size, computing a metabolic interaction potential value for a few species was not computationally feasible. To not miss the potential impact of these species, we established a correlation between the metabolic resource overlap scores in MRS and SCmod media to identify the top five communities with the lowest metabolic resource overlap scores (Fig. 3E).

### *S. boulardii* boosts probiotic bacteria numbers in multi-species co-cultures.

**Figure 4:**
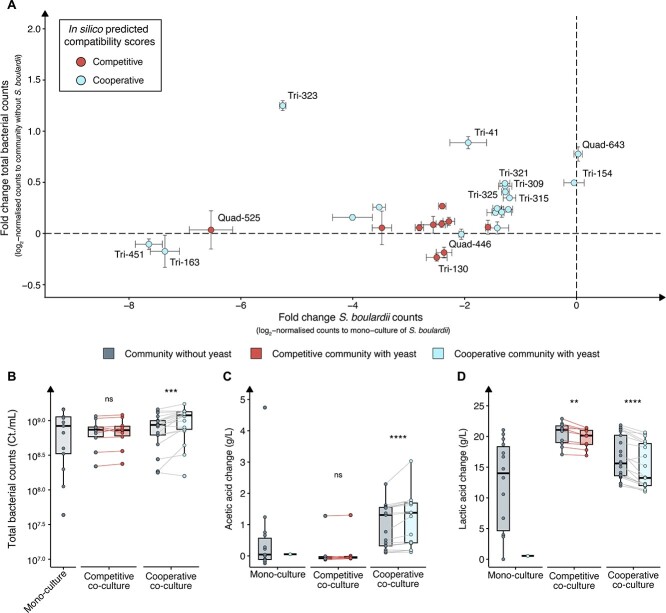
Multi-species co-cultures with and without *S. boulardii*. (A) Scatter plot for visualising bacteria and *S. boulardii* enrichment in multi-species co-culture. Shown in the *y*-axis: log_2_ fold change of total bacterial counts in multi-species co-culture with *S. boulardii* over the total bacterial counts without *S. boulardii and* shown on the *x*-axis: log_2_ fold change of *S. boulardii* counts in multi-species co-culture over the *S. boulardii* mono-culture. Light blue dots signify cooperative multi-species co-cultures anticipated to exhibit strong synergy with *S. boulardii*, based on metabolic interaction potential and metabolic resource overlap scores. Conversely, red dots indicate competitive multi-species co-cultures expected to demonstrate antagonism or reduced efficacy in the presence of *S. boulardii*. Data are presented as the mean of three replicates ±SD. End point values of (B) bacterial counts, and change in (C) acetic acid concentration, and (D) lactic acid concentration between mono-culture and multi-species co-culture with and without *S. boulardii* in MRS media. Basal level of acetic acid (5.13 g/l) and lactic acid concentration (0.30 g/l) were subtracted from each sample to determine the change in concentration. Each data point represents the mean of three replicates from either a mono-culture or a co-culture. Data are presented as the mean of three replicates. *P* values were computed using the Wilcoxon signed rank test. Significance level at *P* < .05, ^*^P < .05, ^*^^*^P < .01, ^*^^*^^*^P < .001, ^*^^*^^*^^*^P < .0001.

After employing the SMETANA *in silico* predictions to guide our selection process, we streamlined the large pool of potential communities and narrowed it down to a feasible number for testing. Specifically, we selected the 10 communities with the highest metabolic community synergy scores (Fig. 3D), five communities with the lowest metabolic resource overlap scores (Fig. 3E), and three communities consisting of species that exhibited strong performance in pairwise co-cultures for further experimental investigation (Cooperative communities; Table 2). Additionally, we selected the top 10 candidates with the most adversely affected by the presence of yeast in terms of metabolic resource overlap and metabolic interaction potential (Competitive communities; Table 2, Supplementary Fig. S10). These selections were made to evaluate the predictive utility of SMETANA towards community function and stability upon a given media. All multi-species cultures were conducted in MRS media to facilitate better bacterial growth (Fig. 2B). The presence of *S. boulardii* in the cooperative predicted communities experimentally exhibited a significant increase of the total bacterial numbers (Fig. 4A and B). In contrast, for the competitive predicted community, no significant benefit was observed with *S. boulardii* present (Fig. 4A and B). In most cases, *S. boulardii* was significantly negatively impacted by the presence of the other bacteria, both in the cooperative and competitive predicted communities (Supplementary Fig. S12). This impact could be a result of the higher bacterial load seen in the communities (Supplementary Fig. S5). Two communities, Tri-154 and Quad-643, stood out, where the total bacterial communities were significantly positively impacted by the *S. boulardii* presence, and *S. boulardii* was insignificantly impacted by the bacteria’s presence (Fig. 4A; Supplementary Fig. S12).

**Table 2:** List of the experimentally tested multi-species communities.

Community ID	Strain 1	Strain 2	Strain 3	Strain 4	Selection criteria
**Cooperative communities**					
Tri-3	*L. gasseri (2)*	*L. reuteri (2)*	*B. longum* subsp. *infantis*		MCS
Tri-41	*L. gasseri (2)*	*L. reuteri (2)*	*L. jensenii (3)*		MCS
Tri-43	*L. gasseri (2)*	*L. reuteri (2)*	*L. buchneri (2)*		MCS
Tri-50	*L. gasseri (2)*	*L. rhamnosus (2)*	*L. jensenii (3)*		MCS
Tri-67	*L. gasseri (2)*	*L. acidophilus (2)*	*L. buchneri (2)*		MCS
Tri-119	*L. reuteri (2)*	*L. jensenii (3)*	*B. longum* subsp. *infantis*		MCS
Tri-154	*B. longum* subsp. *infantis*	*L. crispatus (1)*	*L. brevis (2)*		MRO
Tri-163	*B. longum* subsp. *infantis*	*L. johnsonii (2)*	*L. brevis (2)*		MRO
Tri-294	*L. reuteri (2)*	*L. jensenii (3)*	*L. rhamnosus (2)*		MCS
Tri-309	*L. reuteri (2)*	*L. jensenii (3)*	*L. acidophilus (2)*		MCS
Tri-315	*L. reuteri (2)*	*L. jensenii (3)*	*L. crispatus (1)*		Pairwise
Tri-321	*L. reuteri (2)*	*L. jensenii (3)*	*L. johnsonii (2)*		Pairwise
Tri-322	*L. reuteri (2)*	*L. jensenii (3)*	*L. buchneri (2)*		MCS
Tri-323	*L. reuteri (2)*	*L. jensenii (3)*	*L. delbrueckii (2)*		MCS
Tri-325	*L. reuteri (2)*	*L. jensenii (3)*	*L. brevis (2)*		Pairwise
Tri-451	*L. johnsonii (2)*	*L. salivarius (3)*	*L. brevis (2)*		MRO
Quad-560	*B. longum* subsp. *infantis*	*L. johnsonii (2)*	*L. rhamnosus (2)*	*L. brevis (2)*	MRO
Quad-643	*B. longum* subsp. *infantis*	*L. johnsonii (2)*	*L. buchneri (2)*	*L. brevis (2)*	MRO
**Competitive communities**					
Tri-73	*L. gasseri (2)*	*L. crispatus (1)*	*L. buchneri (2)*		MRO
Tri-105	*B. longum* subsp. *infantis*	*L. paracasei (1)*	*L. rhamnosus (2)*		MIP
Tri-112	*B. longum* subsp. *infantis*	*L. paracasei (1)*	*L. delbrueckii (2)*		MIP
Tri-130	*B. longum* subsp. *infantis*	*L. buchneri (2)*	*L. rhamnosus (2)*		MRO
Tri-164	*B. longum* subsp. *infantis*	*L. buchneri (2)*	*L. delbrueckii (2)*		MRO
Tri-402	*L. buchneri (2)*	*L. crispatus (1)*	*L. acidophilus (2)*		MRO
Tri-430	*L. buchneri (2)*	*L. crispatus (1)*	*L. delbrueckii (2)*		MRO
Quad-446	*B. longum* subsp. *infantis*	*L. paracasei (1)*	*L. rhamnosus (2)*	*L. buchneri (2)*	MIP
Quad-447	*B. longum* subsp. *infantis*	*L. paracasei (1)*	*L. rhamnosus (2)*	*L. delbrueckii (2)*	MIP
Quad-525	*B. longum* subsp. *infantis*	*L. reuteri (2)*	*L. buchneri (2)*	*L. delbrueckii (2)*	MIP

Further investigation of the metabolic profile in the multi-species co-cultivation of bacteria and *S. boulardii* revealed an increase in final acetic acid concentration for the cooperative communities whereas the competitive demonstrated no significant difference (Fig. 4C). Moreover, 14 out of 18 cooperative communities tested, significantly increased their acetic acid production in the presence of *S. boulardii*, whereas none of the competitive communities did (Supplementary Fig. S12). The largest increases were observed in the communities with the best *S. boulardii* growth performance, Tri-154 and Quad-643, with a significant increase of 493 and 734 mg/l respectively, in acetic acid concentration (Fig. 4C, Supplementary Fig. S12). All tested communities exhibited a decrease in lactic acid concentration when *S. boulardii* was added to the community, apart from one community that increased in levels (Fig. 4D, Supplementary Fig. S12).

### *S. boulardii* is a donor of amino acids in multi-species co-cultures.

**Figure 5:**
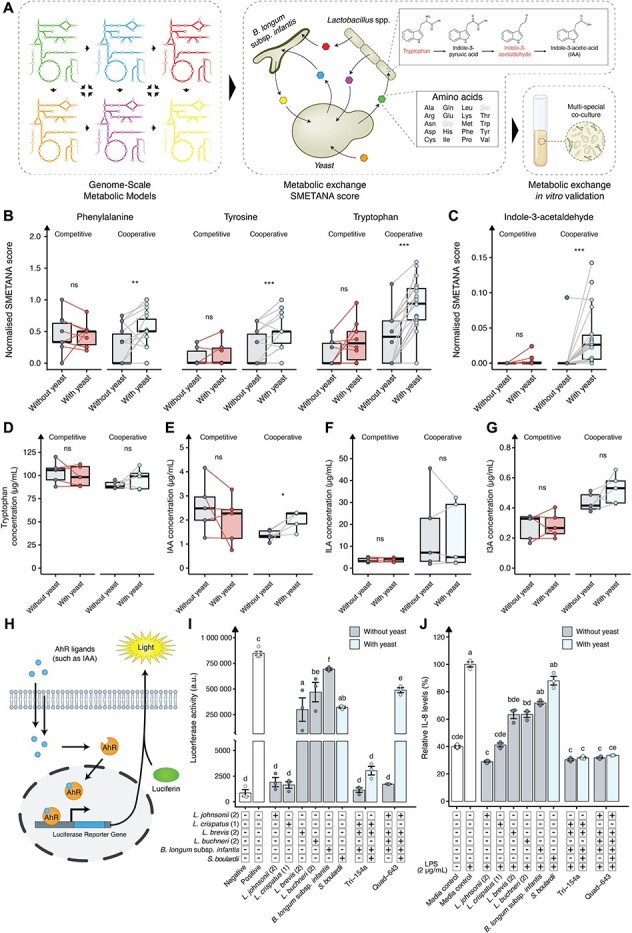
The effect on tryptophan metabolism in multi-species co-cultivation with and without *S. boulardii*. (A) Graphical overview of the framework from genome-scale metabolic modelling predicted metabolite exchange to experimentally validate the production of the metabolites. (B) Normalised SMETANA score of the aromatic amino acids (phenylalanine, tyrosine, and tryptophan) in competitive and cooperative predicted communities with and without yeast. (C) Normalised SMETANA score of the indole-3-acetaldehyde in competitive (red) and cooperative (blue) predicted communities with and without yeast. Experimentally validated (D) tryptophan concentration (μg/ml), (E) IAA concentration (μg/ml), (F) ILA concentration (μg/ml), and (G) I3A concentration (μg/ml) in spent media from a 24-h multi-species co-cultivation in MRS. Competitive communities are coloured red and cooperative communities are coloured blue. Each data point represents the mean of three replicates from either a community with or without *S. boulardii*. (H) Graphical illustration of the mammalian *in vitro* assay, where a luciferase reporter gene is strategically placed downstream of an aryl hydrocarbon receptor (AhR) activator site. Upon the introduction of AhR ligands, such as Indole-3-acetic acid (IAA), and the substrate luciferin, luminescence is produced. The intensity of the emitted light is directly proportional to the concentration of AhR ligands present. (I) Luciferase activity emitted from the AhR reporter cell line exposed to spent media (20% v/v). 20% v/v MRS media was used as the negative control and 20 μM FICZ as the positive control. (J) Relative IL-8 levels in the HT-29 cell line challenged with LPS (2 μg/ml) and either 10% (v/v) MRS media (control; white) or 10% (v/v) spent media with (light blue) and without (grey) *S. boulardii* for 24 h. Data are normalised to media control with LPS and presented as the mean of three replicates ±SEM. Each data point represents a community. *P* values were computed using the Wilcoxon signed rank test for panels (B) and (C); a paired independent two-sample *t*-test was used for panel (D, E, F, and G); and one-way ANOVA with Tukey HSD adjustment for multiple comparisons for panel I and J. Each letter nn above the bars indicates statistically distinct groups. Bars labelled with the same letter indicate no significant difference between those groups, whereas bars labelled with different letters indicate significant differences. Significance level at *P* < .05, ^*^P < .05, ^*^^*^P < .01, and ^*^^*^^*^P < .001.

After identifying that *S. boulardii* improves acetic acid production in bacterial communities, we sought to investigate the broader metabolic dynamics within the community. To identify other metabolic changes, we employed genome-scale metabolic modelling to calculate a SMETANA score of the metabolic exchanges between the communities with and without yeast (Fig. 5A). In addition, we investigated with flux variability analysis, the theoretical yield of amino acids for *S. boulardii* and each community member. Our analysis revealed yeast as a strong contributor of amino acids, which potentially benefits the needs of the bacterial community members for these metabolites (Supplementary Figs S13 and S14, Supplementary Table S5). For all amino acids, except serine and glycine, yeast was a net contributor of amino acids. In the specific context of the Quad-643 community, yeast was responsible for approximately 56.8% of all amino acid donations (Supplementary Fig. S15A), whereas in the Tri-322 community, yeast accounted for 80.8% of the total amino acids supplied to the bacterial members (Supplementary Fig. S15A).

Among all observed changes in amino acid fluxes, a rise in SMETANA score normalised to community size for the aromatic amino acids was observed (Fig. 5B). Given their importance as precursors to key biochemical compounds [62], our investigation was expanded to search for downstream metabolites with predicted increases in fluxes (Supplementary Fig. S14). In the computed multi-species co-culture with yeast, numerous metabolite alterations were observed, but only indole-3-acetaldehyde—linked to the aromatic amino acid pathway—was significantly elevated (Fig. 5C, Supplementary Fig. S14). Indole-3-acetaldehyde is a precursor for indole-3-acetic acid (IAA), which is a known microbial tryptophan derivative to regulate intestinal homeostasis and mitigate inflammatory responses [41, 63]. This finding led us to experimentally quantify levels of tryptophan and IAA, along with the derivatives indole-lactic acid (ILA) and indole-3-carbaldehyde (I3A) in multi-species co-culture in MRS media. We selected the top and bottom five performing communities based on *S. boulardii* counts from the competitive and cooperative. Co-cultivation with *S. boulardii* showed a marginal change in tryptophan levels after 24 h (Fig. 5D). However, IAA levels demonstrated a significant increase of 25% in the cooperative communities (Fig. 5E), with Quad-643 and Tri-154 experiencing increases of 43% and 217%, respectively (Supplementary Fig. S16). No significant changes in ILA or I3A levels were observed (Fig. 5F and G).

To determine the therapeutic relevance of these increases, we conducted a mammalian cell assay using a cell line with a luciferase reporter gene positioned downstream of an Aryl hydrocarbon Receptor (AhR) activation site. When AhR ligands, such as IAA [64], along with the substrate luciferin are introduced, the system generates luminescence. This design facilitates the detection of AhR activation events through a measurable luminescent signal (Fig. 5H). As such we observed a strong activation of the receptor when exposed to the community Quad-643 with *S. boulardii*, resulting in 280-fold higher activation compared to the community without *S. boulardii* (Fig. 5I). Further testing of other communities revealed no consistent trends with the addition of *S. boulardii*, and overall, the communities demonstrated reduced performance compared to many of the individual strains (Fig. 5I, Supplementary Fig. S17). To evaluate if these findings translate to the communities’ anti-inflammatory properties, we assessed the effectiveness of both the single strains and the communities in reducing IL-8 levels in LPS-stimulated HT-29 cells. The Tri-154 and Quad-643 communities, both with and without *S. boulardii*, outperformed most individual strains in reducing IL-8 levels (Fig. 5J). Further testing of the top five cooperative and bottom five competitive communities demonstrated that most communities significantly reduced IL-8 levels (Supplementary Fig. S18). To assess cell viability, which is correlated with lactate dehydrogenase (LDH) levels [65], we measured LDH release after 24 h of incubation with LPS and the spent media. An increase in LDH release was observed in the communities Tri-154 and Quad-643 with *S. boulardii* (Supplementary Fig. S19), indicating increased cytotoxicity. However, it has previously been demonstrated that some probiotic bacteria increase cytotoxicity in the human colon cancer cell line HT-29, as evidenced by increased LDH release [66, 67]. Therefore, further validation is needed to determine whether the increased LDH release is due to anti-carcinogenic properties of these communities or if they pose a general cytotoxic effect.

## Discussion

The use of bacterial communities has gained considerable interest because of the promise of potentially leveraging the diverse and synergistic health benefits of various probiotics [31, 32]. In this study, we sought to build a resilient community of potential probiotic bacteria and *S. boulardii* with increased production of anti-inflammatory effectors [41, 68]. We initially screened 85 different bacterial strains for their ability to co-exist with *S. boulardii* and identified two promising strains, *L. brevis* (2) and *L. crispatus* (1), which demonstrated a symbiotic relationship with *S. boulardii* in pairwise co-cultivation. The combination of *L. brevis* and *S. boulardii* has been previously suggested as favourable, as evidenced by their frequent use in commercial probiotic cocktails [39]. Although pairwise co-cultivation of *S. boulardii* with *L. brevis* (2) resulted in an increased cell count of *L. brevis* (2), this did not translate into a significant effect on IL-8 levels, unlike the co-cultivation with *L. crispatus* (1) and *B. longum* subsp. *infantis*. This suggests that the magnitude of growth increase might play a role in eliciting a measurable impact on IL-8 production. In the case of *L. brevis* (2), the observed increase in growth may not have been substantial enough to trigger a detectable modulation of IL-8 levels.

*L. rhamnosus*, another commonly paired species with *S. boulardii* in commercial probiotics [39], was associated with the competitive communities in our study and demonstrated poor performance when included in communities with *S. boulardii*. This non-symbiotic relationship, both computationally and experimentally validated, aligns with previous findings of antagonistic interactions that reduce their anti-inflammatory benefits [35]. This underscores the necessity for more comprehensive research to evaluate the effectiveness of these combinations in commercial probiotic formulations. However, strain specificity also plays a significant role in performance, as demonstrated by the large differences observed among the various *L. gasseri* and *L. paragasseri* strains tested in the spent media and pairwise co-cultivation experiments. For example, *L. gasseri* (6) exhibited 850-fold lower *S. boulardii* counts compared to *L. gasseri* (3).

A consistent observation across both pairwise and multi-species co-cultivation was the impact of *S. boulardii* on the production of acetic and lactic acids. *S. boulardii*’s ability to alter lactic acid accumulation has previously been demonstrated in the co-cultivation of *S. boulardii* and *Lactobacillales* in coffee brew fermentation [69]. In our observation, we observed a metabolic shift that favoured the production of acetic acid over lactic acid in MRS media, whereas, in SCmod media, the opposite effect was observed, with lactic acid production being predominant. This phenomenon could be attributed to the growth limitations faced by the bacteria in SCmod media. Without *S. boulardii*, the bacteria maintained an average cell count of 10^6.7^ cells/ml, but with *S. boulardii* present, the count increased to 10^7.4^ cells/ml. However, neither scenario matched the growth levels observed in MRS media, where the bacteria reached an average cell count of 10^8.2^ cells/ml. *S. boulardii* reached an average cell count of 10^7.1^ cells/ml in both MRS and SCmod media, indicating that the pairwise co-cultivation in SCmod favoured less symbiotic exchange compared to that in MRS media.

To further investigate these interactions, genome-scale metabolic modelling has been shown to effectively predict competitive and cooperative communities, based on resource competition and metabolic cross-feeding [52, 70]. However, *in silico* metabolic modelling predictions of single species and communities rely on constraint-based modelling assumptions, which primarily assume a steady-state system. This approach allows for feasible metabolic modelling without requiring kinetic reaction parameters of the included metabolic reactions at the genome scale. Consequently, even though metabolic modelling assuming a system at steady-state cannot predict dynamic changes in any given system, it has been effectively used to investigate theoretical interaction and growth potential when challenged with high numbers of possible communities [46, 47, 71]. We employed genome-scale metabolic modelling to design and examine communities consisting of three to four bacterial species, both with and without yeast. Our predictions suggest that yeast generally improves the metabolic interaction potential and reduce the metabolic resource overlap. Additionally, it demonstrated that yeast can be a strong contributor of amino acids to specific *Lactobacillales* members within a microbial community [36] and increase the amino acid exchange in the community. This suggests that *S. boulardii* potentially creates a supportive niche for these bacteria by supplying essential nutrients, thereby facilitating a synergistic interaction within the community. This does not rule out that other species might also compete for these metabolites in more complex environments. Further investigations, such as studies using mouse models and supplementation of well-defined bacterial-yeast cocktails, are warranted.

Initially we started our metabolic modelling investigation with a pool of over 2000 potential communities. By ranking these for their theoretical interaction potential using computational metabolic modelling and subsequent experimental investigation of the most potential communities, we narrowed our focus to 28 communities, which we experimentally tested. Eventually, we identified two top-performing communities (Tri-154 and Quad-643) that demonstrated superior growth for the bacteria while maintaining the *S. boulardii* counts similar to those in mono-culture. These communities also showed a significant increase in acetic acid concentration and improved IAA levels, two relevant anti-inflammatory effectors [41, 60]. Both communities contained *L. brevis* (2) and *B. longum* subsp. *infantis,* two strains that performed well in the pairwise co-cultivation. Additionally, *L. crispatus* (1) from the pairwise co-cultivation was present in one of the communities. This suggests that these three strains exhibit in general good synergy with *S. boulardii* not only in pairwise co-culture but also in more complex multi-species co-culture. However, both *B. longum* subsp*. infantis* and *L. crispatus* (1) were also present in poorly performing communities, indicating the importance of the other members within the communities.

The Quad-643 community demonstrated the strongest AhR activation change when *S. boulardii* was present. Within the Quad-643 community, each species cultured alone, apart from *L. johnsonii*, showed a high level of receptor activation. However, when these species were combined in the absence of *S. boulardii*, this receptor activation was entirely abolished. Introducing *S. boulardii* restored the community’s receptor activation to a level similar to that observed in the mono-cultures. This suggests that a low-activating strain may either dominate or metabolise the activating substrate in the community. Although neither the communities with *S. boulardii* nor those without significantly outperformed several single strains in activating the AhR, microbial communities exhibit greater resilience and stability against environmental fluctuations and pathogen invasions [72–74]. The functional redundancy within these communities ensures that the loss of one strain does not significantly impact overall performance. Furthermore, the broad range of microbes enables communities to produce and metabolise a wider variety of substrates efficiently, providing diverse beneficial functionalities [74]. Here, we demonstrated that although single strains activated the AhR more effectively, none of the individual strains in Tri-154 and Quad-643 reduced IL-8 levels as much as the community. This highlights the strength of a community with broad functionalities, as evidenced by Quad-643 with *S. boulardii*, which effectively activates the AhR and reduces IL-8 levels. These observations highlight *S. boulardii*’s role in enhancing synergistic interactions and its potential anti-inflammatory properties. However, a limitation of our study is that, even though we assessed *S. boulardii* and the overall bacterial community dynamics in the multi-species co-cultures, we did not evaluate the specific contributions and impacts of individual bacterial strains within these communities. This approach may overlook the nuanced interactions and specific roles of each strain, necessitating further investigations to fully define the roles of individual strains in multi-species communities.

The lack of a strong correlation between tryptophan metabolic derivatives and AhR activation suggests that IAA is not the only ligand activating AhR, aligning with the broad range of ligands described in the literature capable of interacting with this receptor [64, 75, 76]. This observation underscores the importance of methodically characterising probiotic combinations to mitigate the risk of antagonistic interactions among microbial species and highlights the complexity of microbial metabolite interactions with receptors. Further testing is required to understand the mechanisms of the reduction and activation of the AhR, underscoring the need for broader research to fully understand the diverse mechanisms through which microbial communities influence host health mediated by AhR signalling.

Although the underlying mechanisms and interactions warrant further elucidation, and *in vivo* validation is necessary, we demonstrate how the integration of computational analyses with experimental validation can effectively narrow down a vast array of potential probiotic candidates to select a few communities with enhanced anti-inflammatory capabilities. Throughout this study, we underscore the critical role of incorporating *S. boulardii* in designer probiotic communities to enhance synergistic interactions and potential anti-inflammatory properties. Our findings also highlight the importance of strain specificity and the necessity for systematic characterisation of probiotic combinations to optimise their therapeutic potential.

## Supplementary Material

Supplementary_Figures_3rdRevised_wrae212

Table_S1_wrae212

Table_S2_wrae212

Table_S3_wrae212

Table_S4_wrae212

Table_S5_wrae212

Table_S6_wrae212

## Data Availability

The experimental data underlying this article will be shared on reasonable request to the corresponding author*.* All codes and data to reproduce our *in silico* simulations are available at github: https://github.com/mohammadmirhakkak/S_boulardii_bacterial_communities.
