# Supplementary material for: Saccharomyces boulardii enhances anti-inflammatory effectors and AhR activation via metabolic interactions in probiotic communities
Source: ISME J. 2024 Nov 3;18(1):wrae212. doi: 10.1093/ismejo/wrae212 (PMC11631509; doi:10.1093/ismejo/wrae212)
Supplement: Supplementary_Figures_3rdRevised_wrae212 [file supplementary_figures_3rdrevised_wrae212.docx]

**Supplementary Information:**

***Saccharomyces boulardii* enhances anti-inflammatory effectors and AhR activation via metabolic interactions in probiotic communities**

**Running title: *S. boulardii* in probiotic communities**

Authors: Karl Alex Hedin^1^, Mohammad H. Mirhakkak^2^, Troels Holger Vaaben^1^, Carmen Sands^1^, Mikael Pedersen^3^, Adam Baker^4^, Ruben Vazquez-Uribe^1,5^, Sascha Schäuble^2^, Gianni Panagiotou^2,6,7,8,*^, Anja Wellejus^4,*^, Morten Otto Alexander Sommer^1,*^

^1. Novo Nordisk Foundation Center for Biosustainability, Technical University of Denmark, 2800 Kgs. Lyngby, Denmark^

^2. Department of Microbiome Dynamics, Leibniz Institute for Natural Product Research and Infection Biology - Hans Knöll Institute (Leibniz-HKI), 07745 Jena, Germany^

^3. National Food Institute, Technical University of Denmark, 2800 Kgs. Lyngby, Denmark^

^4.^ ^Human Health Biosolutions, Novonesis, 2970 Hørsholm, Denmark,^

^5. Center for Microbiology, VIB-KU Leuven, 3001 Leuven, Belgium^

^6. Friedrich Schiller University, Institute of Microbiology, Faculty of Biological Sciences, 07743 Jena, Germany.^

^7. Friedrich Schiller University, Jena University Hospital, 07743 Jena, Germany.^

^8. Department of Medicine, University of Hong Kong, Hong Kong SAR, China.^

^*Corresponding authors^

^Morten Otto Alexander Sommer, DTU Biosustain, Novo Nordisk Foundation Center for Biosustainability, Technical University of Denmark, Building 220, Søltofts Plads, 2800 Kgs. Lyngby, Denmark. Email:^ [^moas@bio.dtu.dk^](mailto:moas@bio.dtu.dk)

^Anja Wellejus, Human Health Biosolutions, Novonesis, Kogle Alle 6, 2970 Hørsholm, Denmark. Email:^ [^anjwe@novonesis.com^](mailto:anjwe@novonesis.com)

^Gianni Panagiotou, Department of Microbiome Dynamics, Leibniz Institute for Natural Product Research and Infection Biology - Hans Knöll Institute, Adolf-Reichwein-Straße 23, 07745 Jena, Germany. Email:^ [^Gianni.Panagiotou@leibniz-hki.de^](mailto:Gianni.Panagiotou@leibniz-hki.de)

**Supplementary figures:**

**
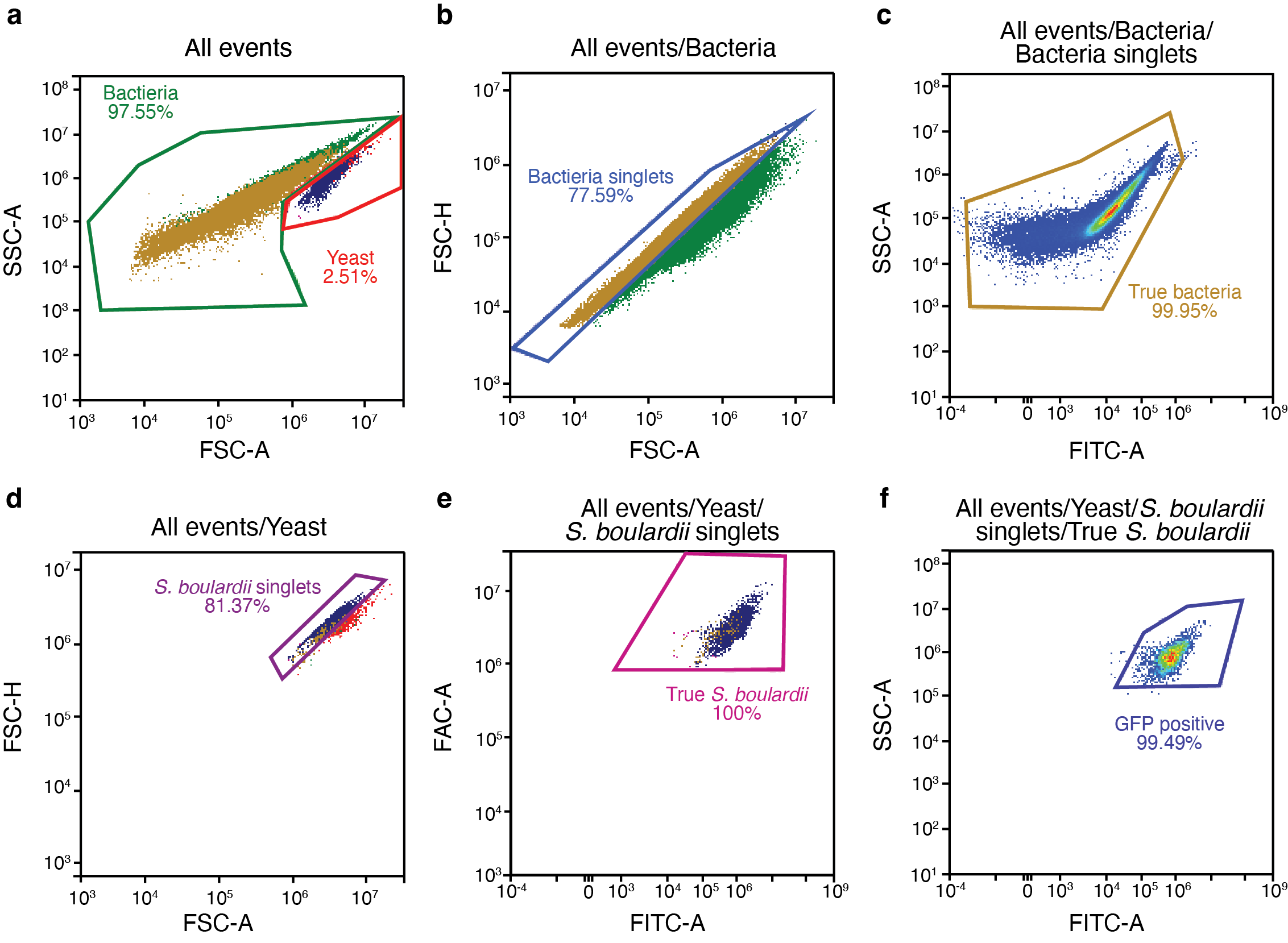
**

**Figure S1: Representative images of the gating strategy for the co-cultures.** **a.** All events, where Bacteria is gated in green box and yeast in red box. **b.** Bacteria singlets are gated from the Bacteria subpopulation. **c.** Total events of Singlet Bacteria **d.** *S. boulardii* singlets are gated from the yeast subpopulation. **e.** Total events of Singlet yeast **f.** GFP positive events taken from the Singlets yeast subpopulation. The same gates were applied to all samples.

**
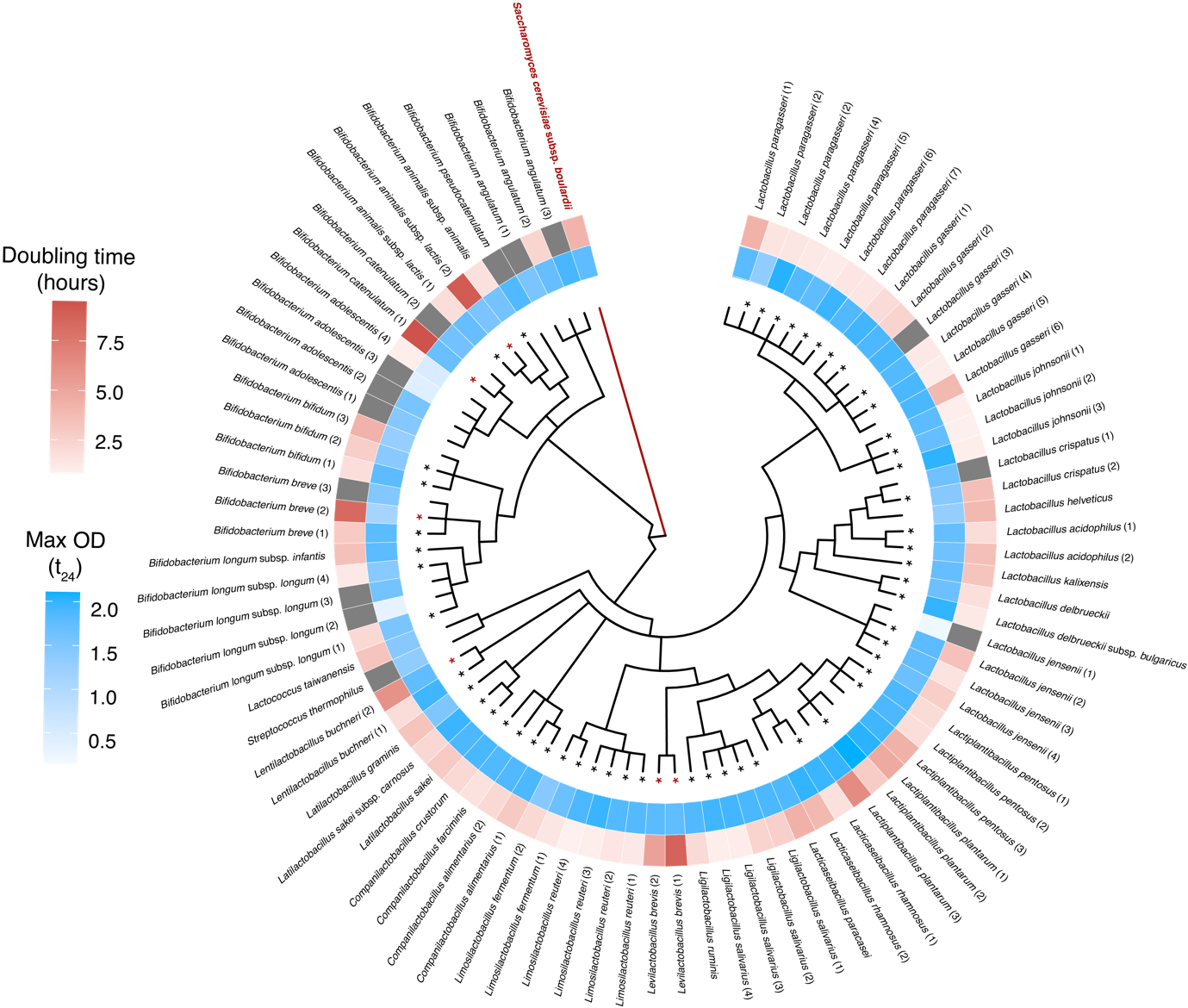
**

**Figure S2. Growth characterisation of the 85 bacterial strains.** Max OD_600_ and growth rate in doubling time (hours) for the 85 bacterial strains and *S. boulardii* (highlighted in red). Data presented as mean of three replicates. Grey boxes indicated doubling time were unable to be calculated. *P* values were computed using independent two sample t-test and adjusted for multiple comparison with false discovery rate with *S. boulardii* as comparison group. Significance level at *P* < 0.05.

**
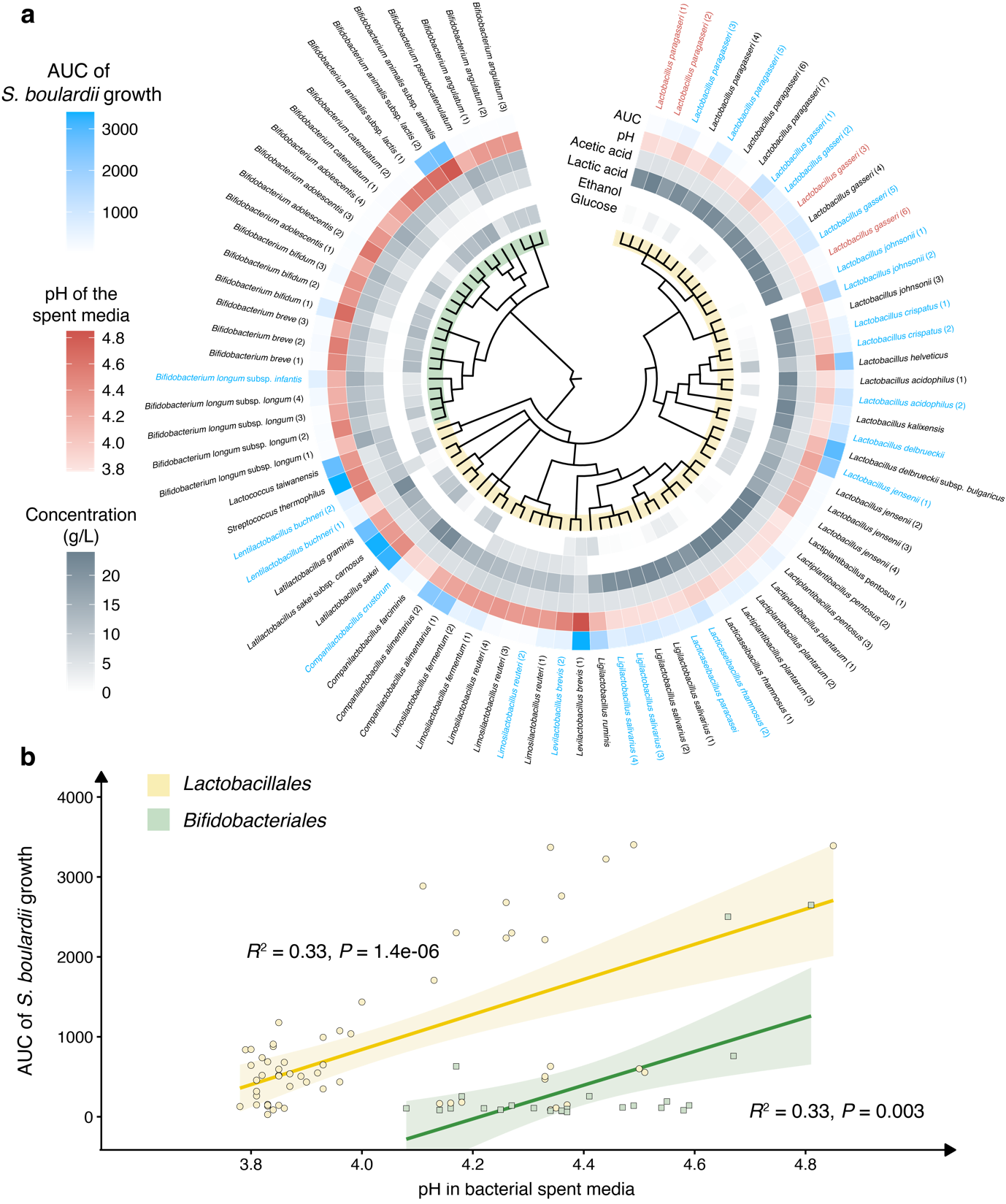
**

**Figure S3. *S. boulardii* growth performance on non-neutralised bacterial spent media. a.** *S. boulardii* growth performance presented as area under the curve (AUC), pH and concentration (g/L) of acetic acid, lactic acid, ethanol, and glucose in the generated spent media. Phylogenetic tree was generated by phyloT v2 based on NCBI Taxonomy, were cooperative (blue) and competitive (red) strain from neutralised spent media are highlighted. **b.** *S. boulardii* growth performance correlated with the pH of the bacterial spent media. Circle yellow data points show *Lactobacillales* and square green data points show *Bifidobacteriales*. The dark yellow/green line indicates the best fit as determined by least square linear regression analysis with Pearson correlation, the yellow/green shaded area indicate the 95% confidence interval. Significance level at *P* < 0.05.

**
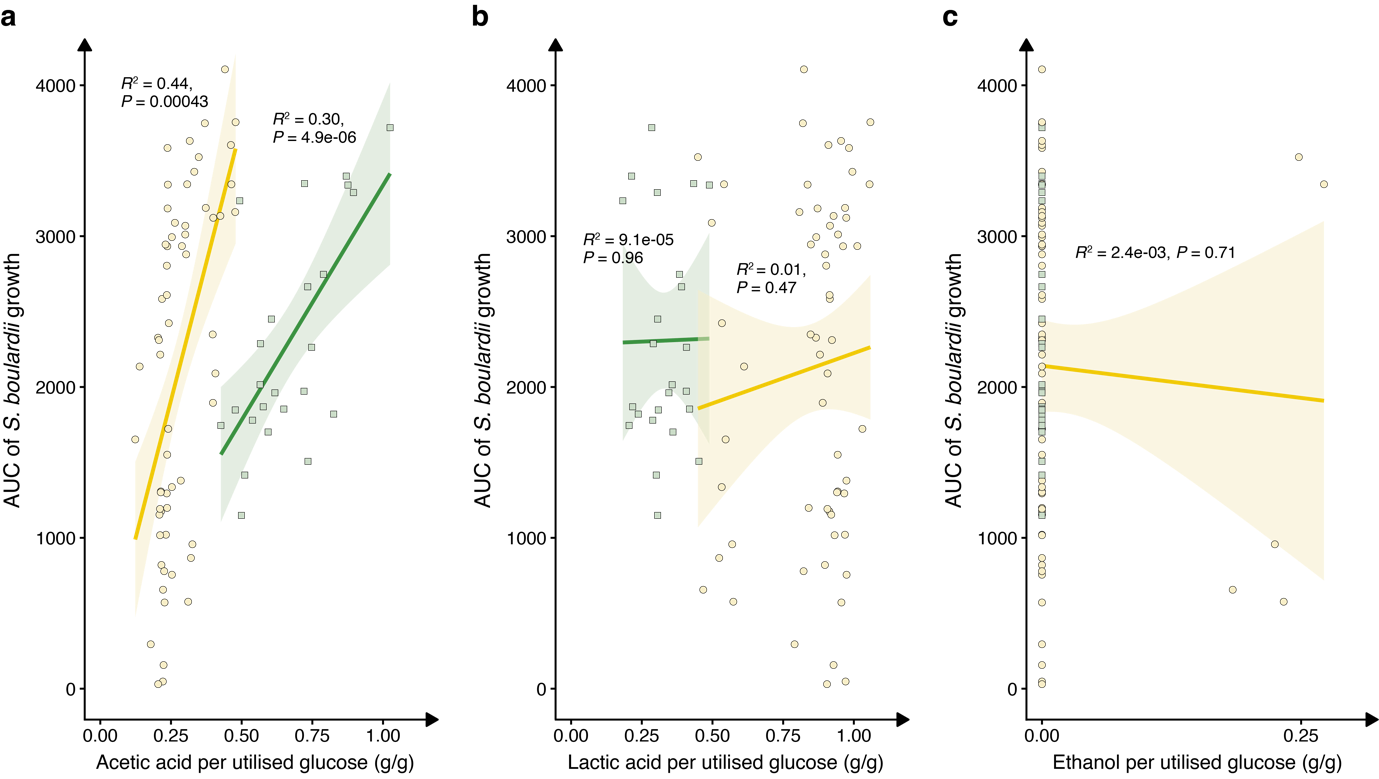
**

**Figure S4. Correlation between S. boulardii growth and the production of organic acids.** Correlation plot of *S. boulardii* growth performance (AUC) correlated to **a.** acetic acid concentration per utilised glucose (g/g), **b.** lactic acid concentration per utilised glucose (g/g), and **c.** ethanol concentration per utilised glucose (g/g). Each data point represents the mean of three replicates. Circle yellow data points show *Lactobacillales* and square green data points show *Bifidobacteriales*. The dark yellow/green line indicates the best fit as determined by least square linear regression analysis with Pearson correlation, the yellow/green shaded area indicate the 95% confidence interval. Significance level at *P* < 0.05.

**
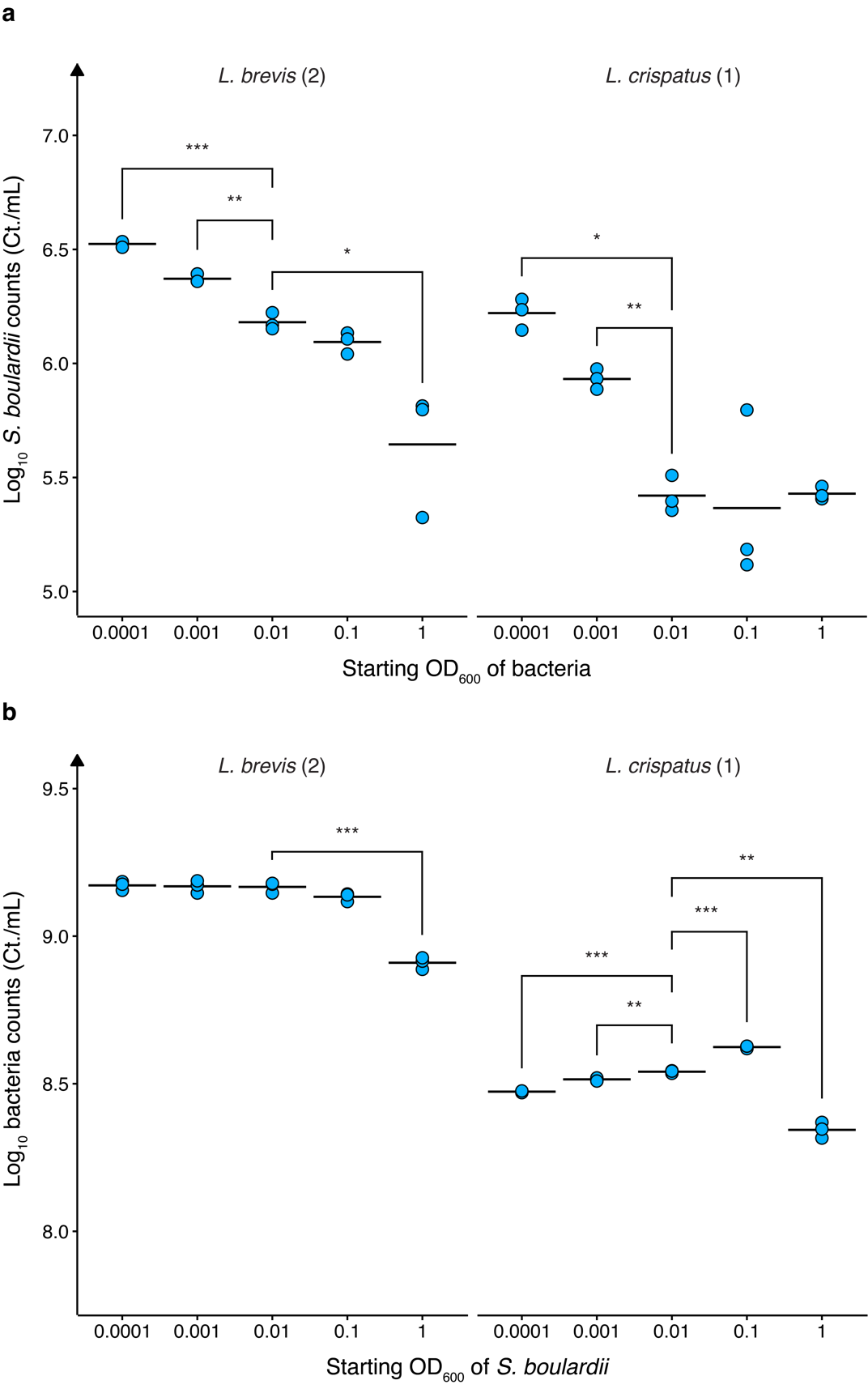
**

**Figure S5. Pairwise co-cultivation of *S. boulardii* and bacteria with different inoculation ratios. a.** Log_10_ *S. boulardii* counts in pairwise co-cultivation with *L. brevis* (2) and *L. crispatus* (1) under varying starting OD_600_ values for bacteria (0.0001, 0.001, 0.01, 0.1, and 1.0), while maintaining a consistent starting OD_600_ of 0.01 for *S. boulardii.* **b.** Log_10_ bacterial counts in pairwise co-cultivation with *L. brevis* (2) and *L. crispatus* (1) under varying starting OD_600_ values for *S. boulardii* (0.0001, 0.001, 0.01, 0.1, and 1.0), while maintaining a consistent starting OD_600_ of 0.01 for the bacteria. *P* values were computed using independent two sample t-test and adjusted for multiple comparison with false discovery rate. Significance level at *P* < 0.05. * *P* < 0.05, ** *P* < 0.01 and *** *P* < 0.001.

**
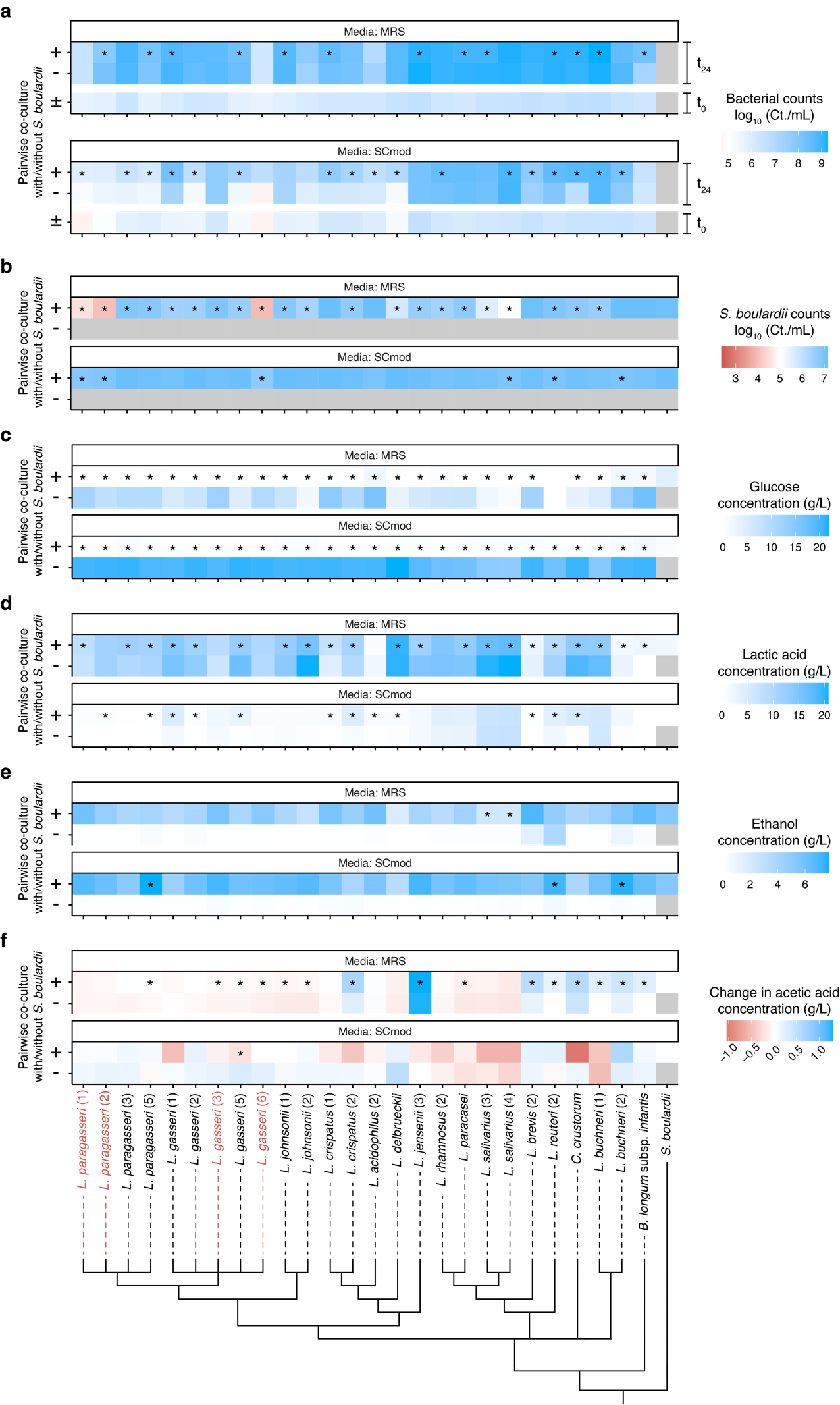
**

**Figure S6. Pairwise co-culture of *S. boulardii* and bacterial strains. a.** Bacterial counts (log_10_ Ct./mL) and **b.** *S. boulardii* counts (log_10_ Ct./mL) in mono-cultures and pairwise co-cultures with and without *S. boulardii* in MRS and SCmod. Initial t_0_ *S. boulardii* count was quantified to 4.96 log_10_ Ct./mL and 5.25 log_10_ Ct./mL in MRS and SCmod, respectively. End point values of **c.** glucose, **d.** lactic acid and **e.** ethanol concentration (g/L) in mono-cultures of bacteria and pairwise co-cultures with *S. boulardii* in MRS and SCmod. **f.** Change in acetic acid concentration (g/L) in mono-cultures of bacteria and pairwise co-cultures with *S. boulardii* in MRS and SCmod. Data presented as mean of three replicates. Strains coloured red are the four bacteria candidates that exhibited inhibitory effects on *S. boulardii* growth. *P* values were computed using independent two sample t-test and adjusted for multiple comparison with false discovery rate. In panel a, c, d, and f, the respective pairwise co-cultures’ mono-culture is set as reference group. In panel b and e, *S. boulardii* mono-culture is set as reference group. Significance level at *P* < 0.05.


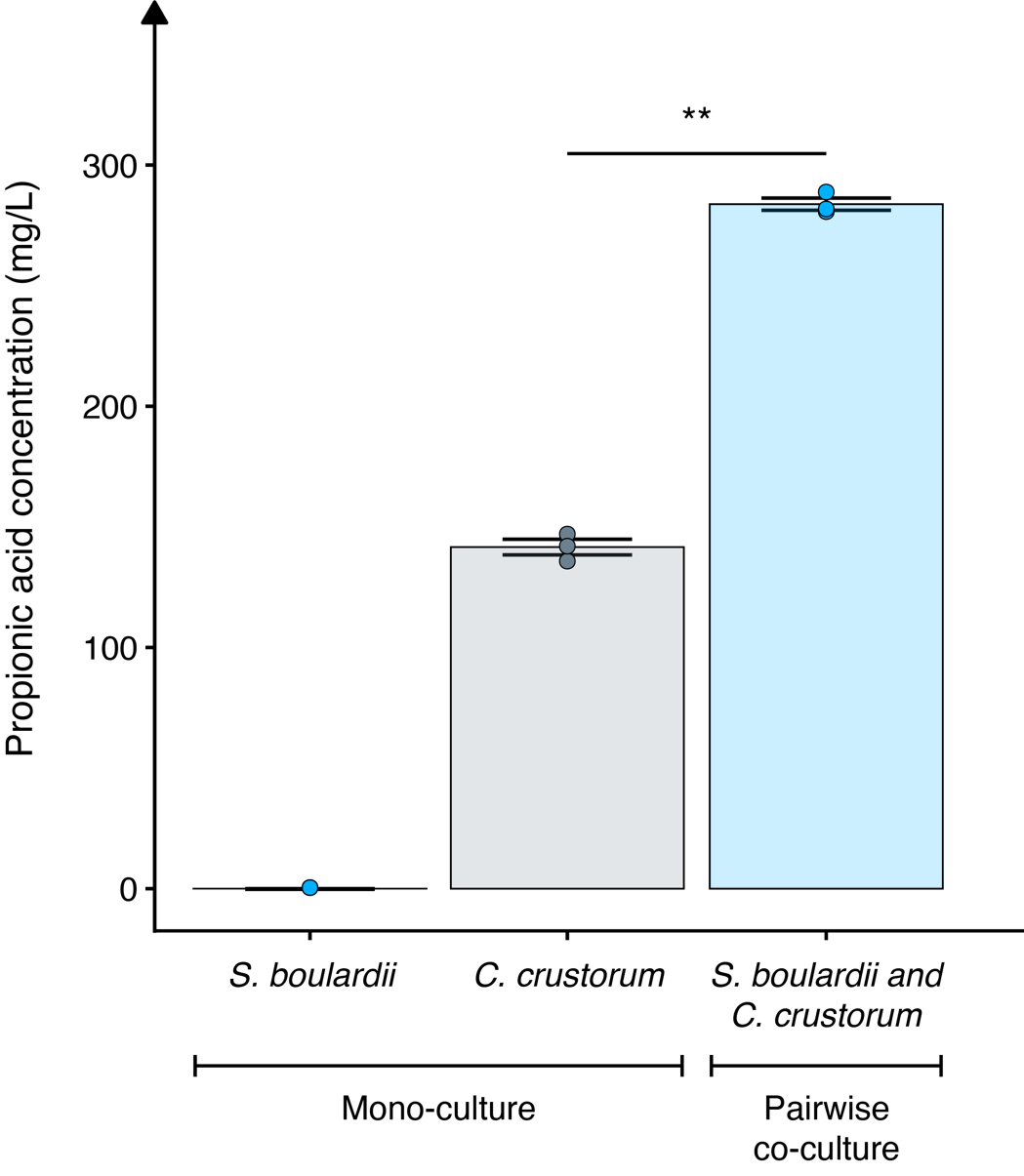


**Figure S7. Propionic acid quantification.** End point values of propionic acid concentration (mg/L) in the harvested supernatant from mono-culture of *S. boulardii* and *C. crustorum*, and pairwise co-culture of *S. boulardii* and *C. crusorum* in MRS media. Basal level of propionic acid in MRS was determent to 0 mg/L. Limit of detection 100 mg/L. Data presented as mean of three replicates. *P* values were computed using independent two sample t-test. Significance level at *P* < 0.05. ** *P* < 0.01

**
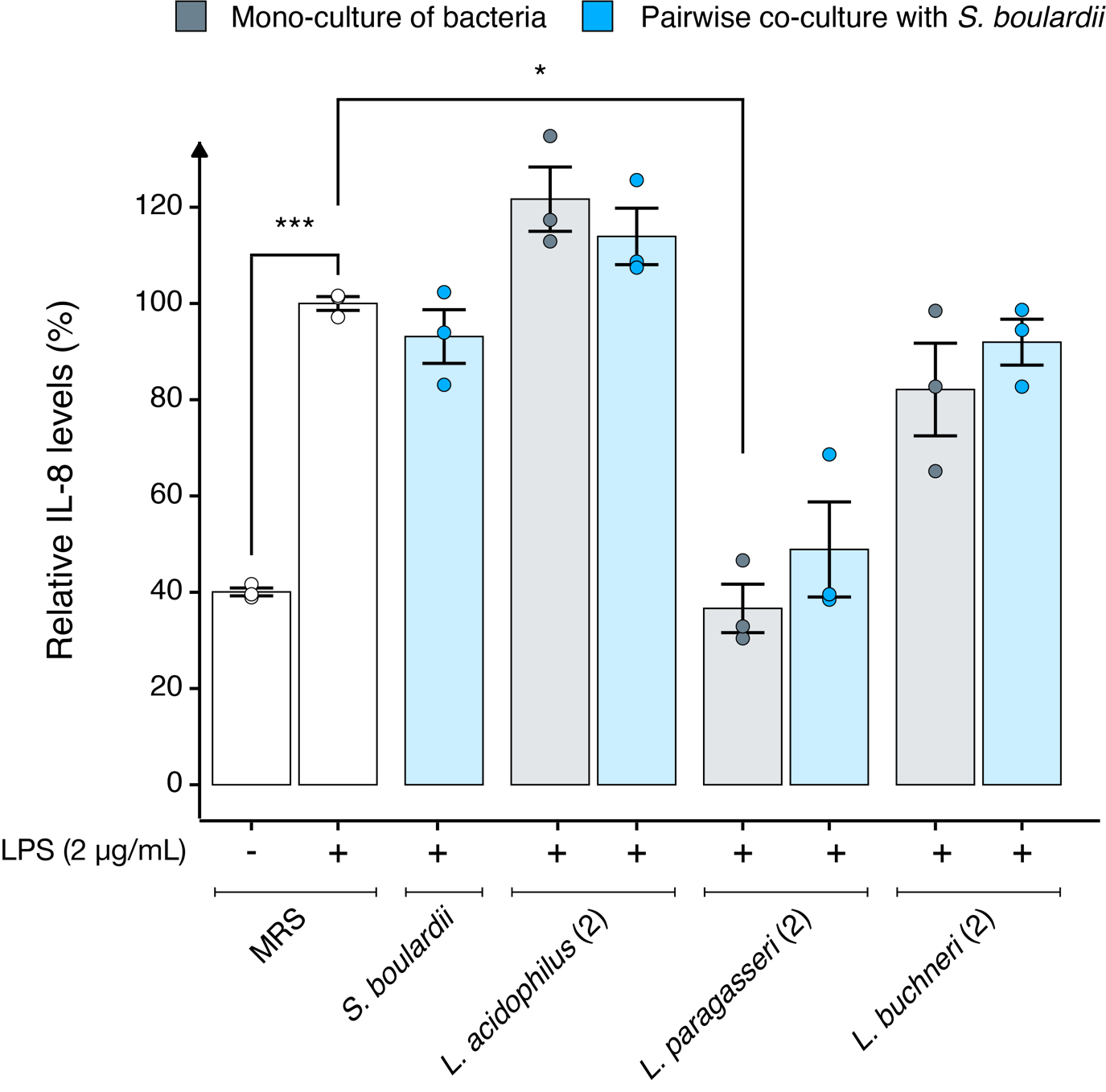
**

**Figure S8: Anti-inflammatory properties of pairwise co-culture and mono-culture of the three communities with *S. boulardii* resulting in largest bacteria.** Relative IL-8 production in the HT-29 cell line challenged with LPS (2 µg/mL) and either 10% (v/v) MRS media (control; white) or 10% (v/v) spent media with (blue) and without (grey) *S. boulardii* for 24 hours. Data is presented as the mean of three replicates ± SEM. *P* values were computed using independent two sample t-test and adjusted for multiple comparison with false discovery rate with MRS + LPS as reference. Significance level at *P* < 0.05. * *P* < 0.05, ** *P* < 0.01 and *** *P* < 0.001.

**
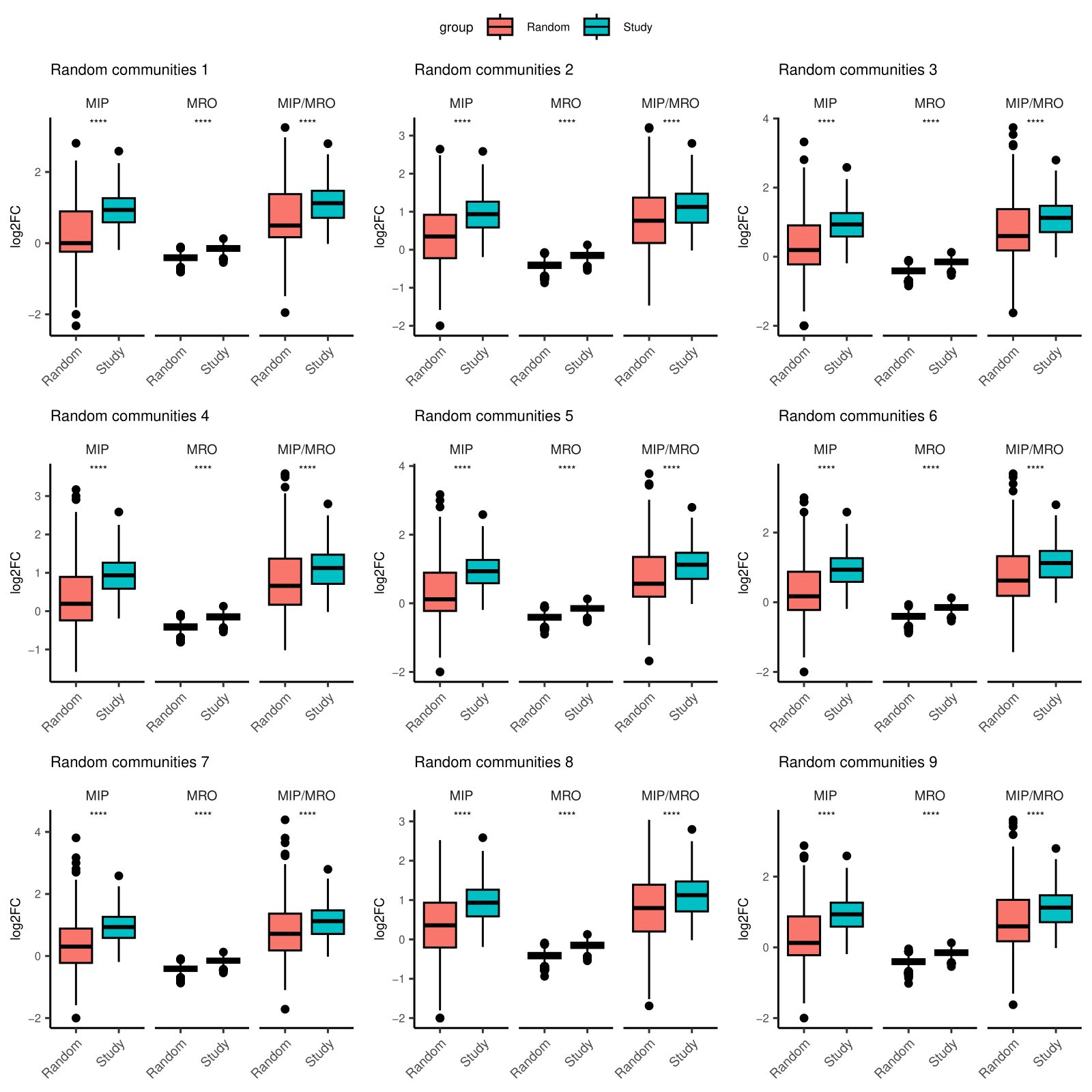
**

**
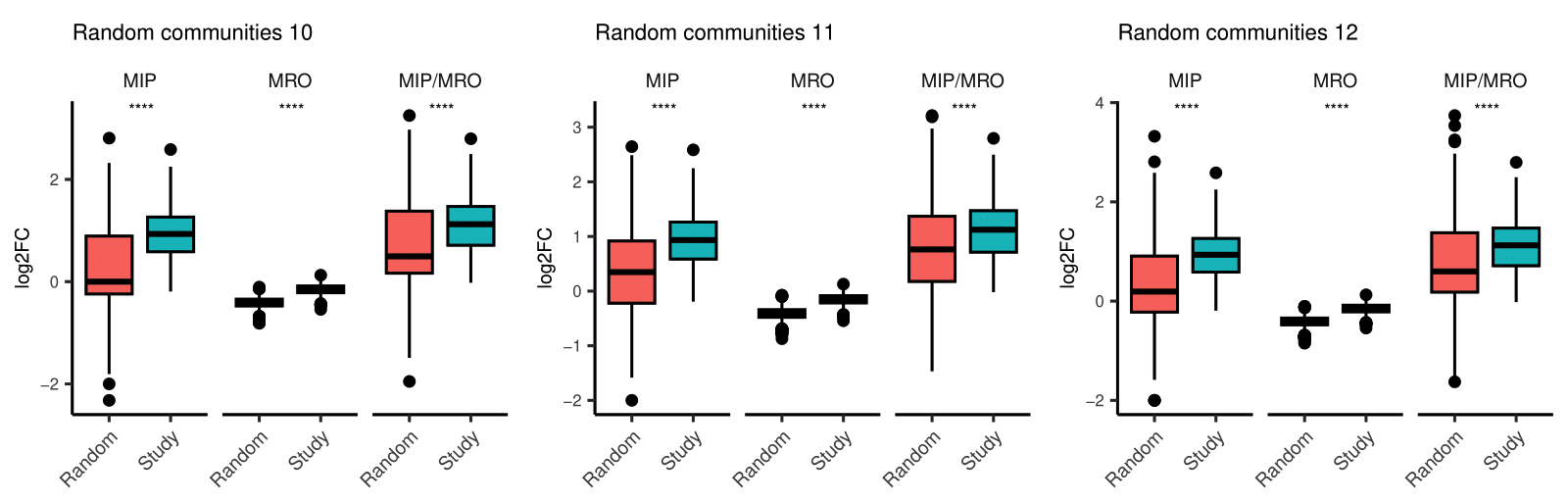
**

**
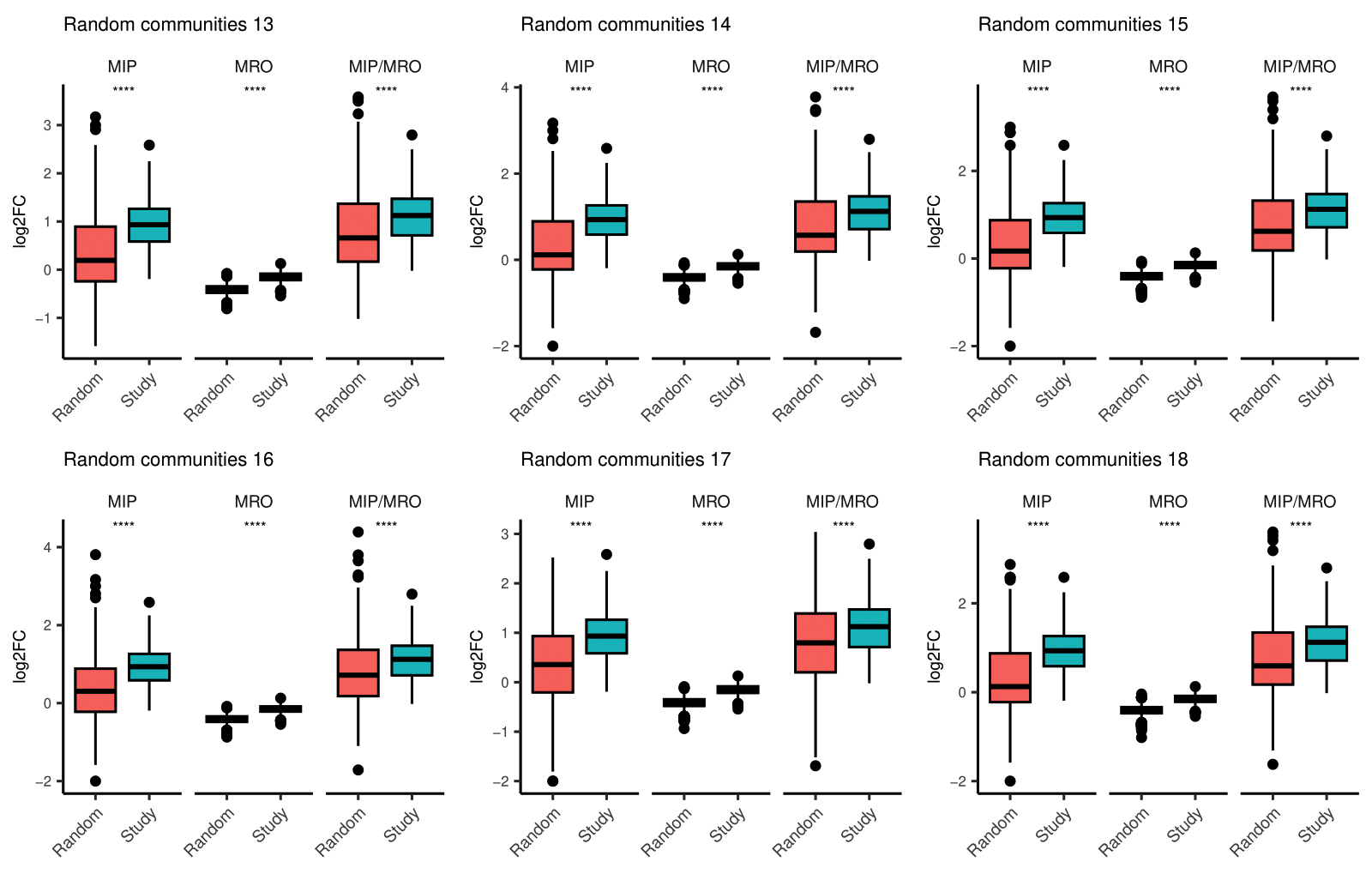
**

**
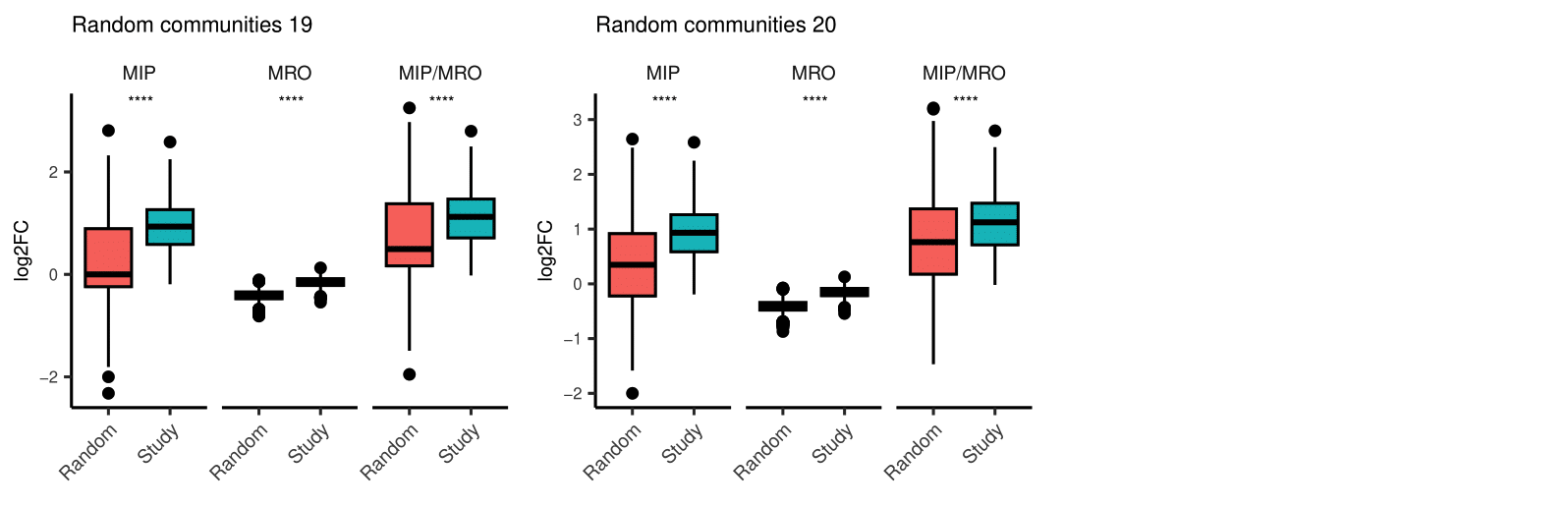
**

**Figure S9.** **Change of SMETANA cooperation and competition scores of the bacterial communities upon presence of a fungal model.** Log_2_ fold changes of MIP, MRO, and MIP/MRO (overall synergy score) (derived from SMETANA in a complete medium) of the bacterial communities with one fungal model over the bacterial communities without any fungal model for 20 sets of random communities (with and without a random fungi) vs. studied bacterial communities (with and without *S. boulardii*). *P* values were computed using Wilcoxon signed rank test. Significance level at *P* < 0.05. * *P* < 0.05, ** *P* < 0.01, *** *P* < 0.001, and **** *P* < 0.0001

**
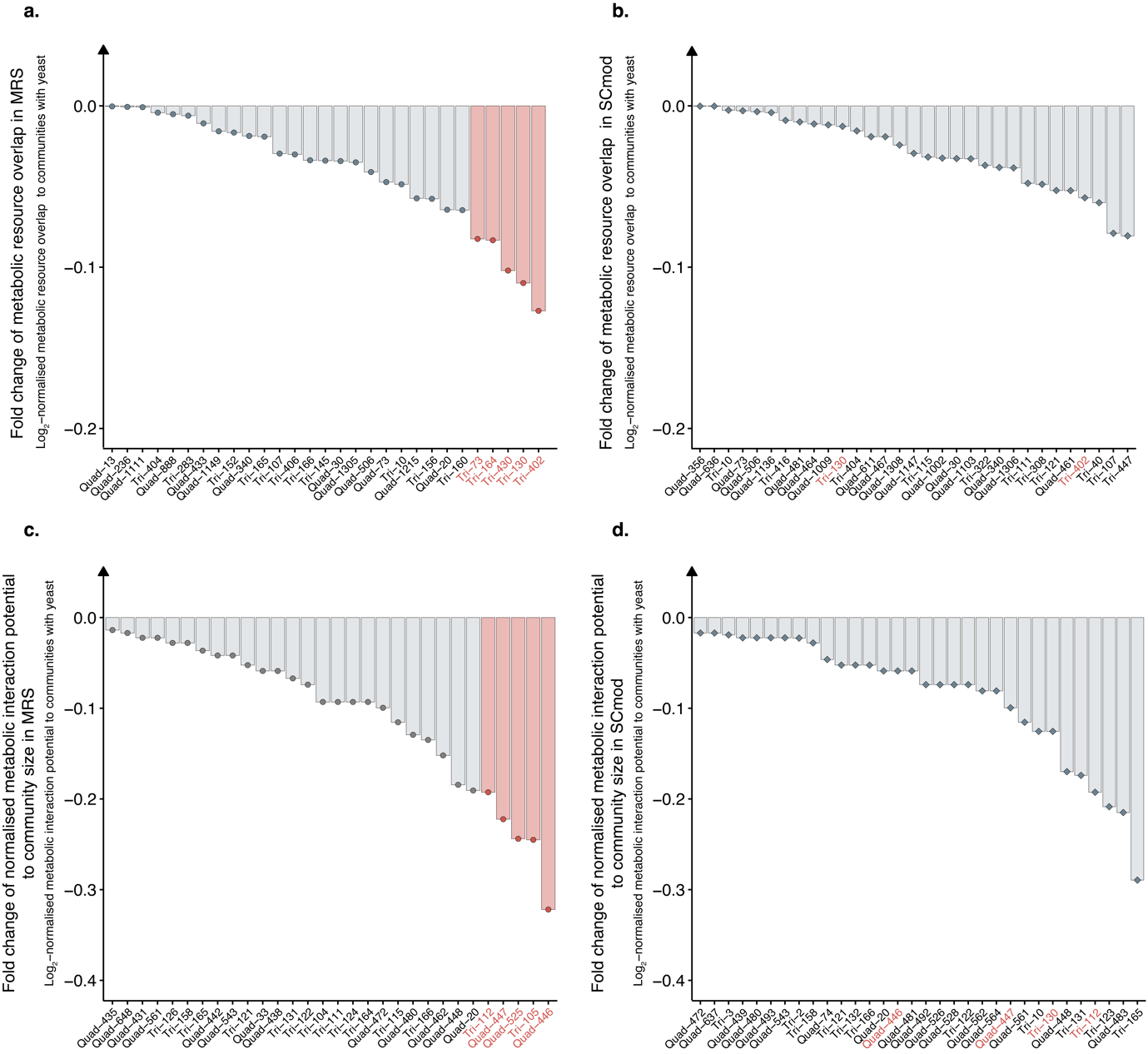
**

**Figure S10. Communities computationally predicted to be negatively impacted by yeast presence.** Fold change in metabolic resource overlap score, presented as log2-normalised, for (a) MRS media and (b) SCmod media, relative to the metabolic resource overlap score in communities without yeast. Data is shown only for communities with a negative impact. The most competitive communities (bottom 5 with the greatest negative impact in MRS media) are highlighted in red. Fold change in metabolic interaction potential score, normalised to community size, for (c) MRS media and (d) SCmod media, log2-normalized relative to the metabolic interaction potential score in communities with yeast. Data is shown only for communities with a negative impact. The most competitive communities (bottom 5 with the greatest negative impact in MRS media) are highlighted in red.

**
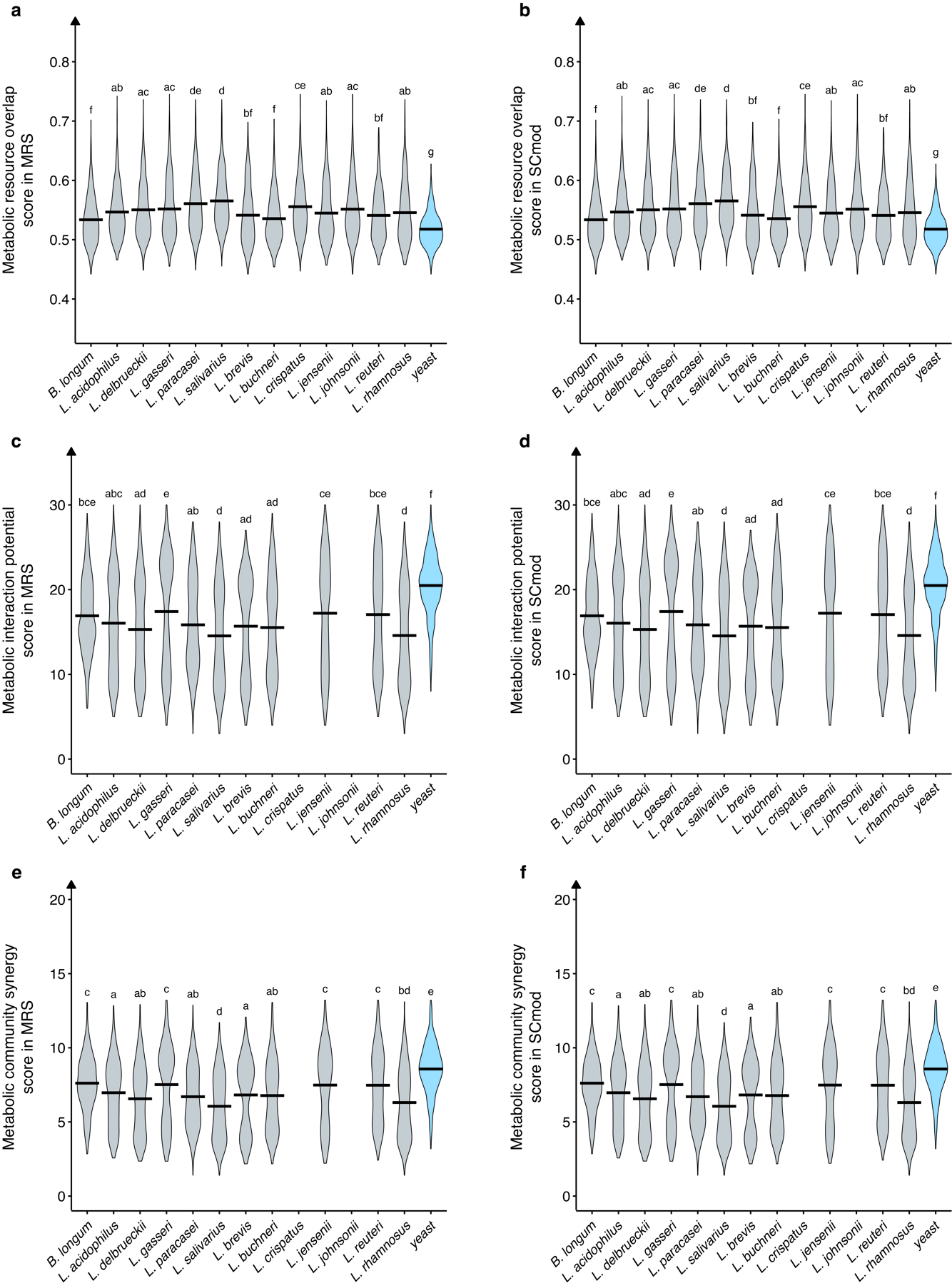
**

**Figure S11. Species metabolic interaction analysis.** Violin plot of metabolic resource overlap score **a.** in MRS and **b.** in SCmod. Violin plot of metabolic interaction potential score **c.** in MRS and **d.** in SCmod. Violin plot of metabolic community synergy score **e.** in MRS and **f.** in SCmod. *P* values were computed using One-way ANOVA with Tukey HSD adjustment for multiple comparison. Each letter (a, b, c, d, e, f, and g) above the bars indicates statistically distinct group. Bars labelled with the same letter indicate no significant difference between those groups, while bars labelled with different letters indicate significant differences. Significance level at *P* < 0.05.

**
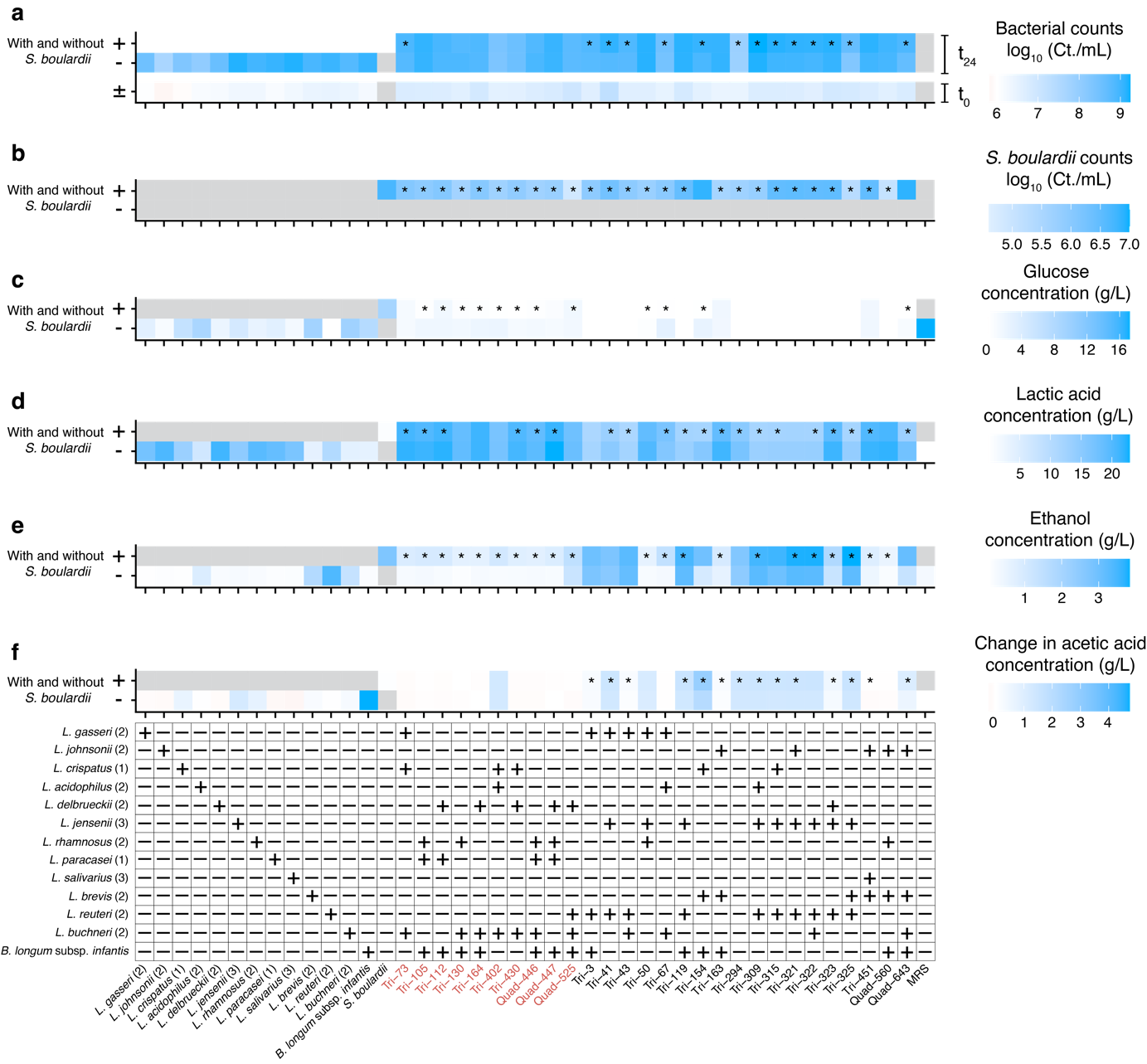
**

**Figure S12. Multi-special co-culture of *S. boulardii* and bacterial strains. a.** Bacterial and **b.** *S. boulardii* counts in the mono-culture and multi-special co-cultures with and without *S. boulardii.* Initial t_0_ *S. boulardii* count was quantified to 5.00 log_10_ Ct./mL. **c.** Glucose, **d.** lactic acid, **e.** ethanol, and **f.** acetic acid concentration changes in the harvest supernatant from respective mono-cultures and multi-special co-culture with and without *S. boulardii.* Data presented as mean of three replicates. Red legend indicates competitive communities. *P* values were computed using independent two sample t-test and adjusted for multiple comparison with false discovery rate. In panel a, c, d, and f, the respective pairwise co-cultures’ mono-culture is set as reference group. In panel b and e, *S. boulardii* mono-culture is set as reference group. Significance level at *P* < 0.05.

**
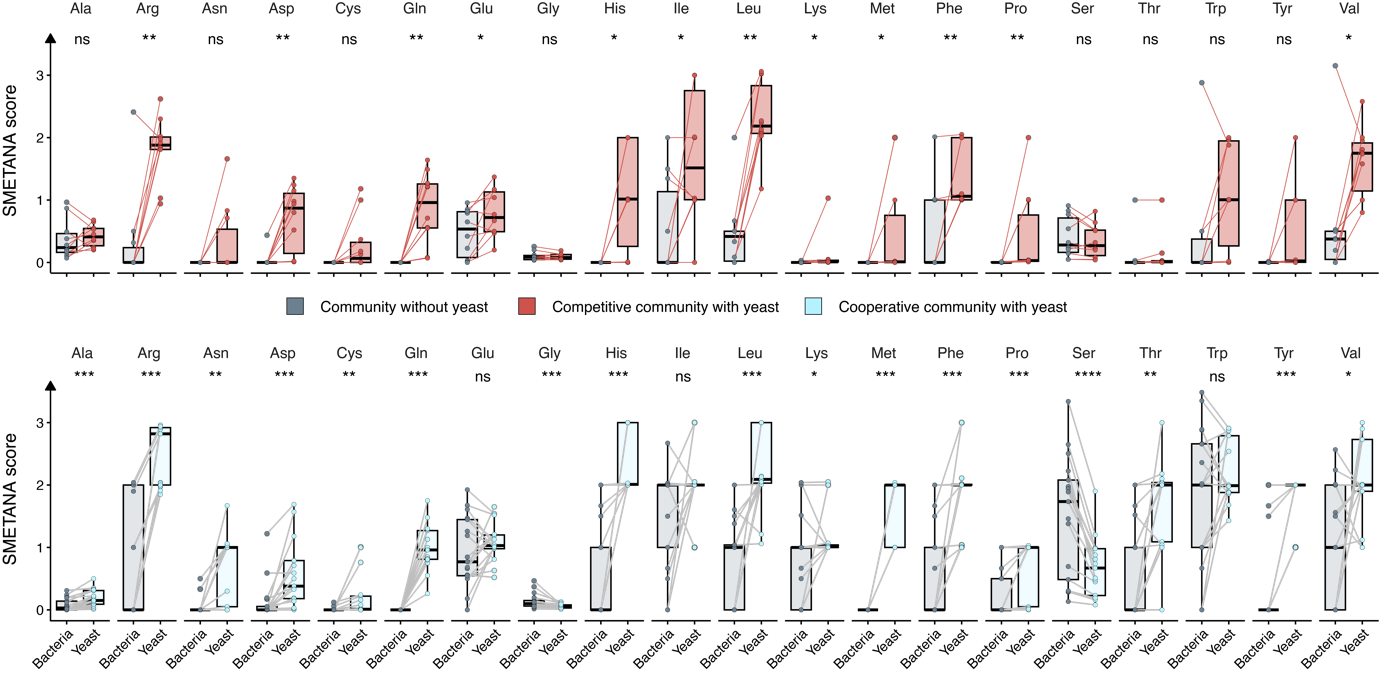
**

**Figure S13. Comparative analysis of amino acid donation by yeast and bacteria within each community.** The bacterial SMETANA score is calculated as the summation of SMETANA scores for each individual bacterium present in the community. Blue coloured communities indicated cooperative communities. Red coloured communities indicated competitive communities. *P* values were computed using Wilcoxon signed rank test. Significance level at *P* < 0.05. * *P* < 0.05, ** *P* < 0.01, *** *P* < 0.001, and **** *P* < 0.0001

**
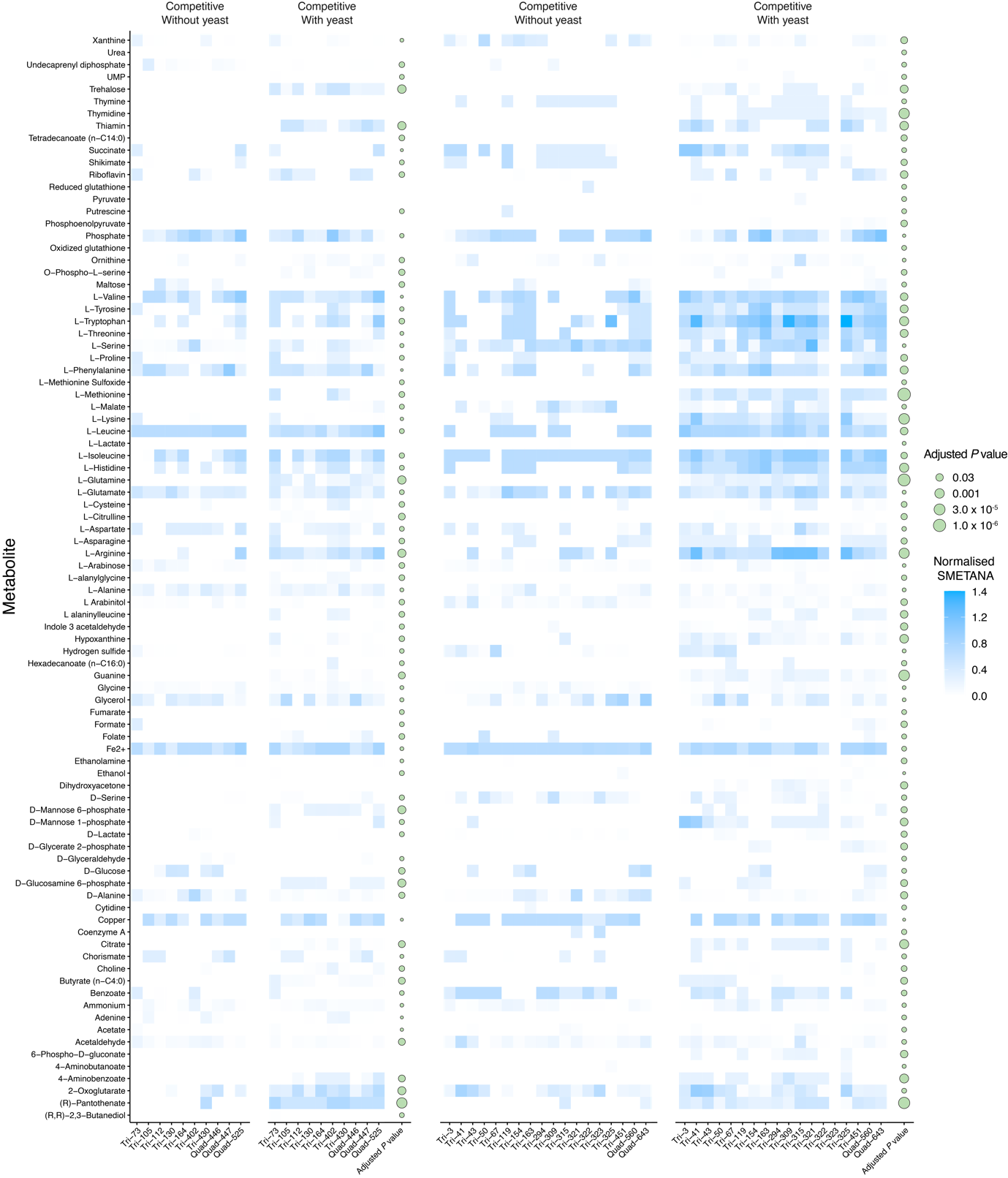
**

**Figure S14. Comparative analysis of all metabolite’s changes.** Computed normalised SMETANA score for used metabolites in the genome-scale metabolic modelling in competitive and cooperative communities with and without yeast. Green circles indicate significance differences between communities with and without yeast. Size of the circle indicate the level of significance. *P* values were computed using independent two sample t-test and adjusted for multiple comparison with false discovery rate. Significance level at *P* < 0.05.

**
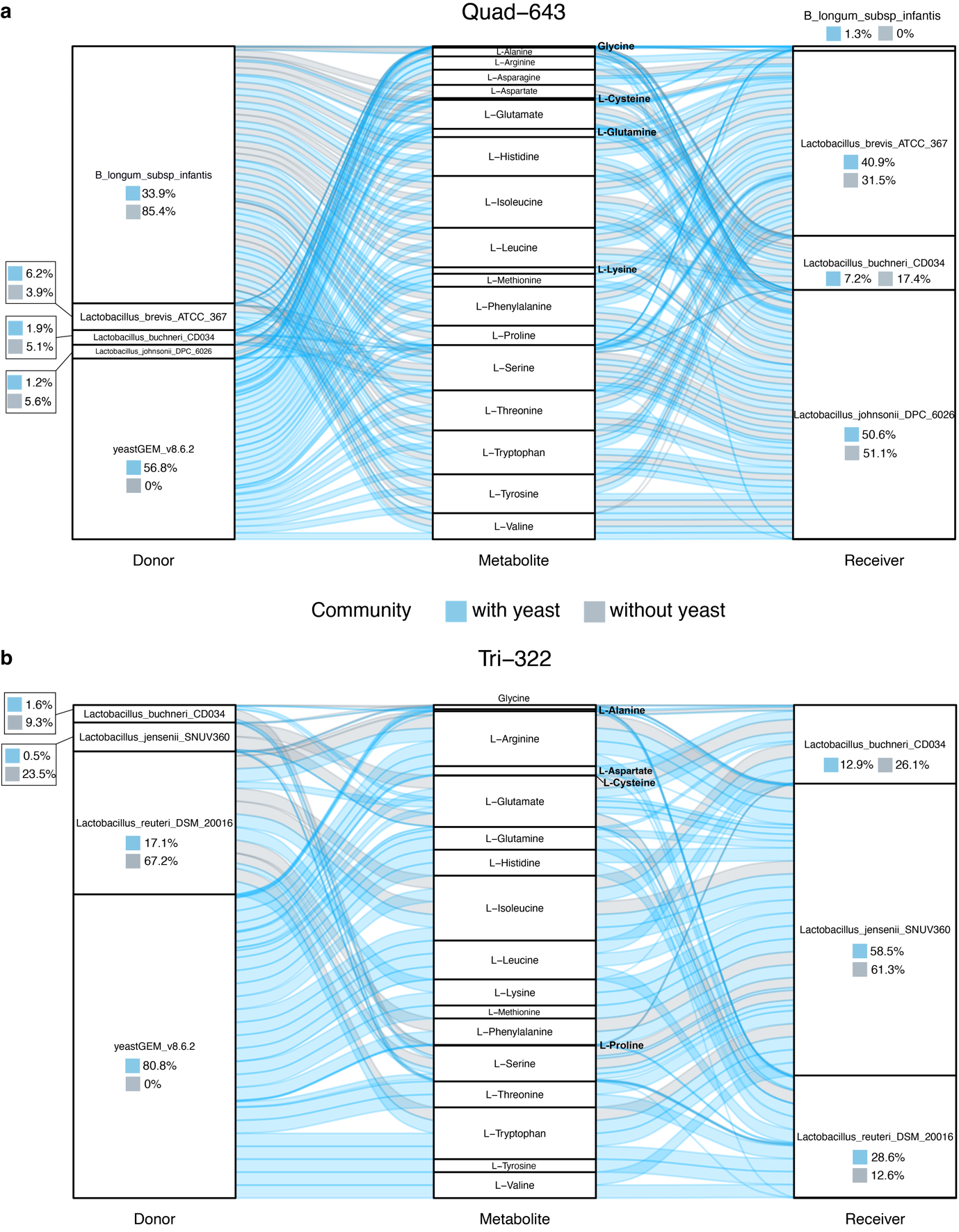
**

**Figure S15. Amino acids changes between different species within the communities. a**. Quad-643. **b.** Tri322. Grey shows the community without yeast. Light blue shows the community with yeast. The percentage is calculated as the ratio of the total SMETANA score (both for donor and receiver).

**
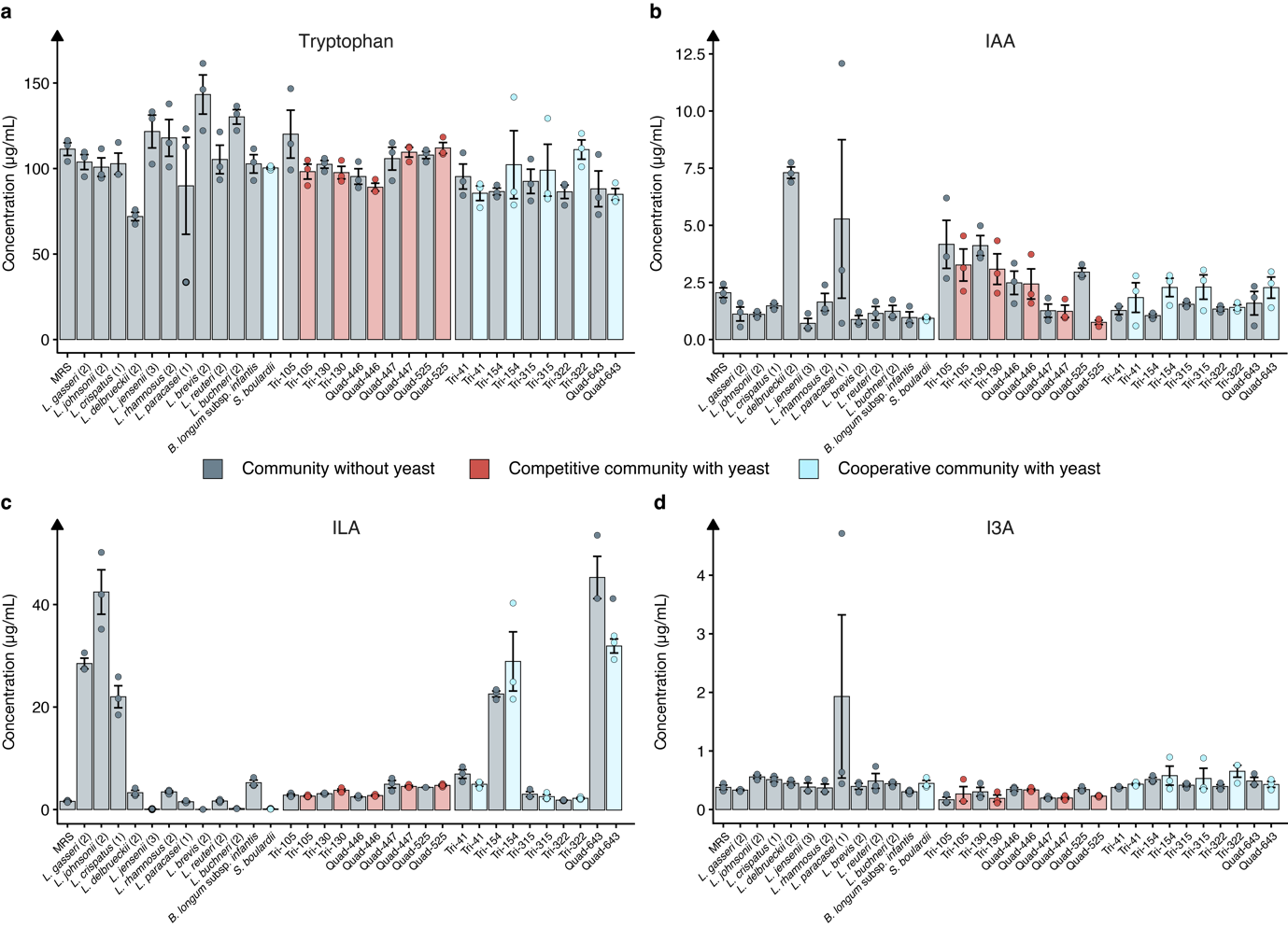
**

**Figure S16. Quantitative levels of tryptophan derivative.** Experimental validated **a.** tryptophan concentration (µg/mL), **b.** IAA concentration (µg/mL), **c.** ILA concentration (µg/mL), and **d.** I3A concentration (µg/mL) in spent media from a 24 hour multi-special co-cultivation of five cooperative communities (light blue) and five competitive communities (red). Data presented as mean of three replicates ± SEM.

**
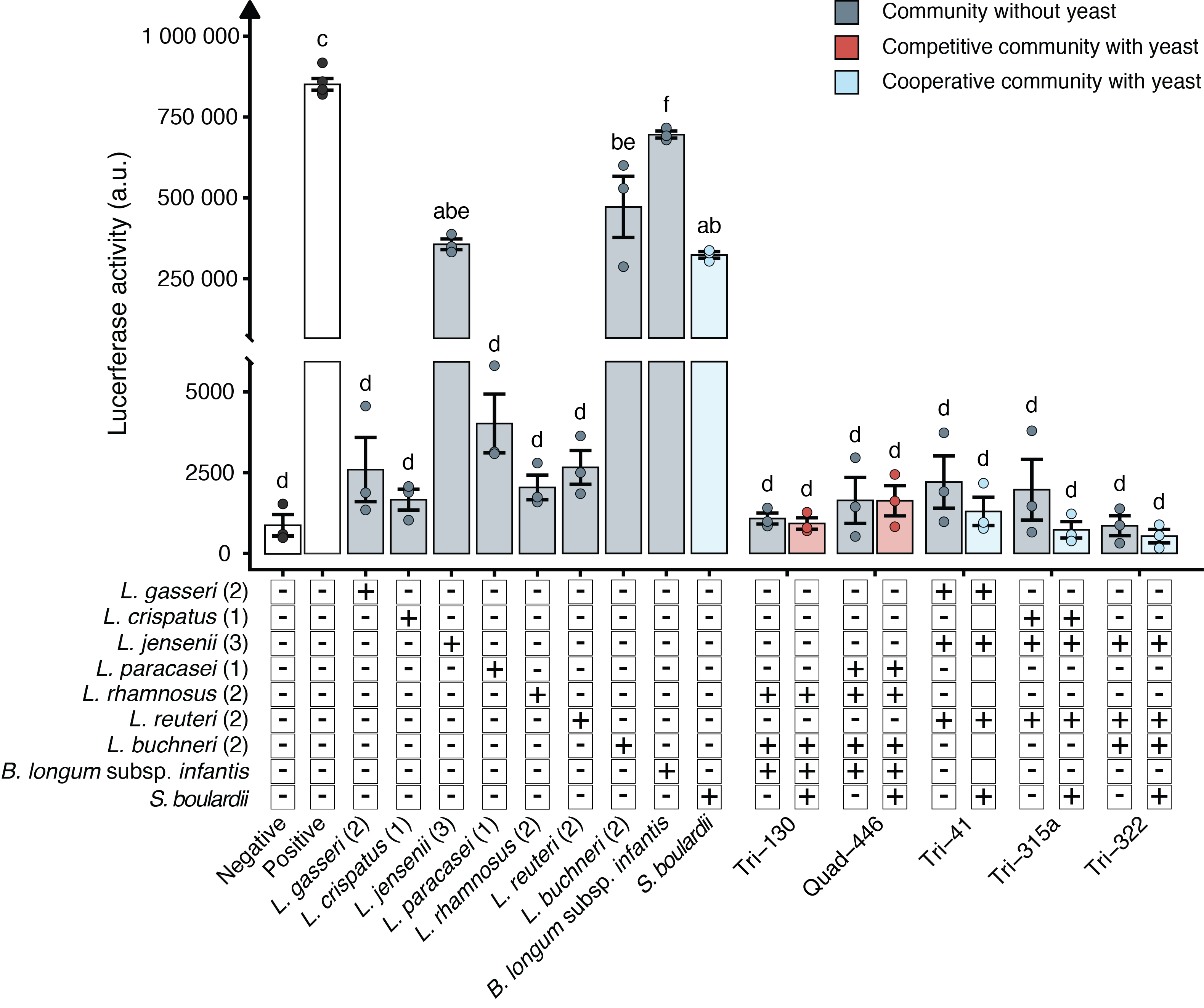
**

**Figure S17. Anti-inflammatory properties of the top five competitive and top three cooperative (excluding Tri-154 and Quad-643) communities** Luciferase activity emitted from the AhR reporter cell line exposed to spent media (20 % v/v). 20 % v/v MRS media was used as the negative control and 20 µM FICZ as the positive control. Each data point represents a community. *P* values were computed using One-way ANOVA with Tukey HSD adjustment for multiple comparisons. Each letter (a, b, c, d, e, and f) above the bars indicates statistically distinct group. Bars labelled with the same letter indicate no significant difference between those groups, while bars labelled with different letters indicate significant differences. Significance level at *P* < 0.05.

**
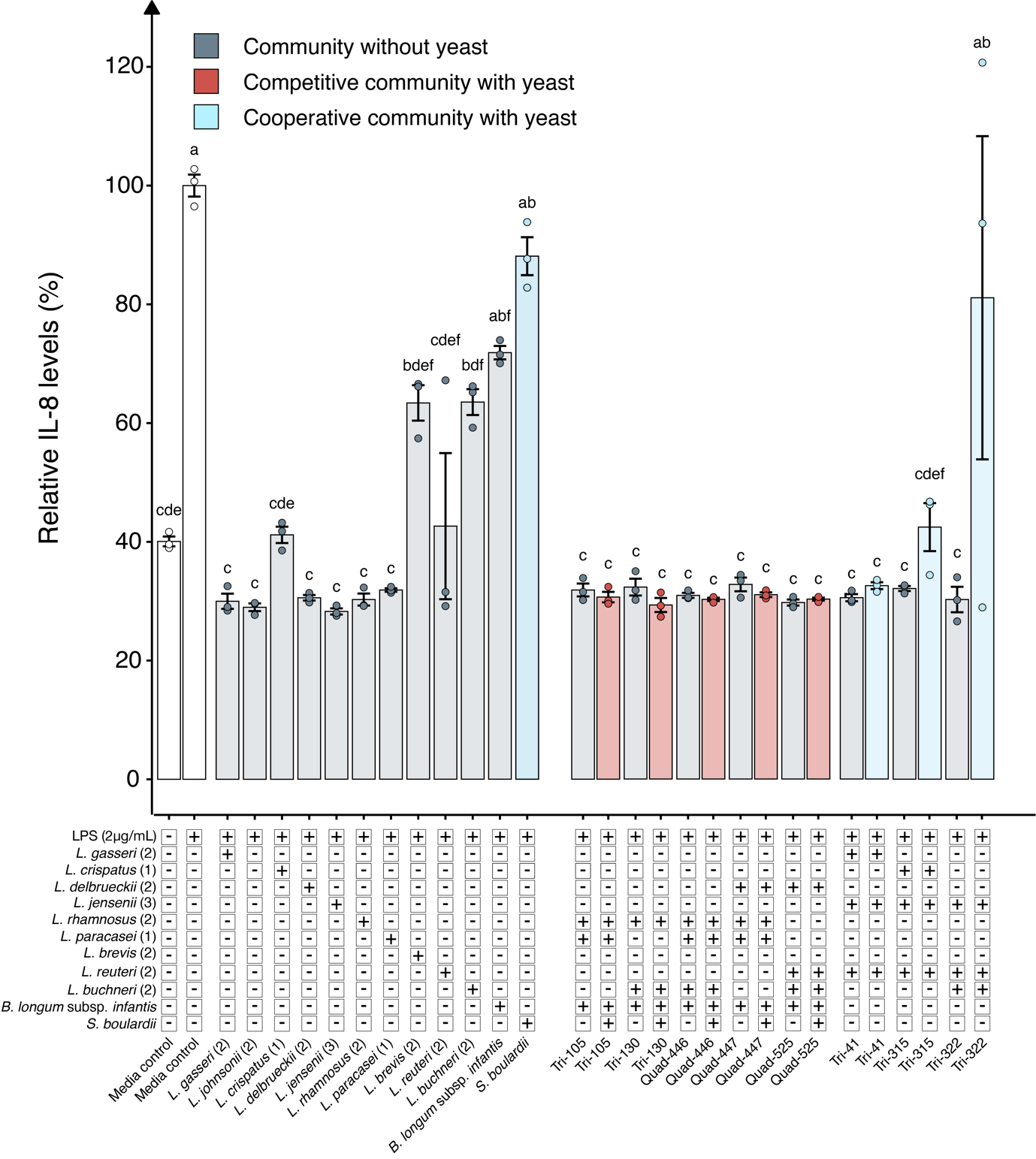
**

**Figure S18. Anti-inflammatory properties of the top five competitive and top three cooperative (excluding Tri-154 and Quad-643) communities.** Relative IL-8 production in the HT-29 cell line challenged with LPS (2 µg/mL) and either 10% (v/v) MRS media (control; white) or 10% (v/v) spent media with (blue) and without (grey) *S. boulardii* for 24 hours. Data is normalised to media control with LPS. Data is presented as the mean of three replicates ± SEM. Each data point represents a community. *P* values were computed using One-way ANOVA with Tukey HSD adjustment for multiple comparisons. Each letter (a, b, c, d, e, and f) above the bars indicates statistically distinct group. Bars labelled with the same letter indicate no significant difference between those groups, while bars labelled with different letters indicate significant differences. Significance level at *P* < 0.05.

**
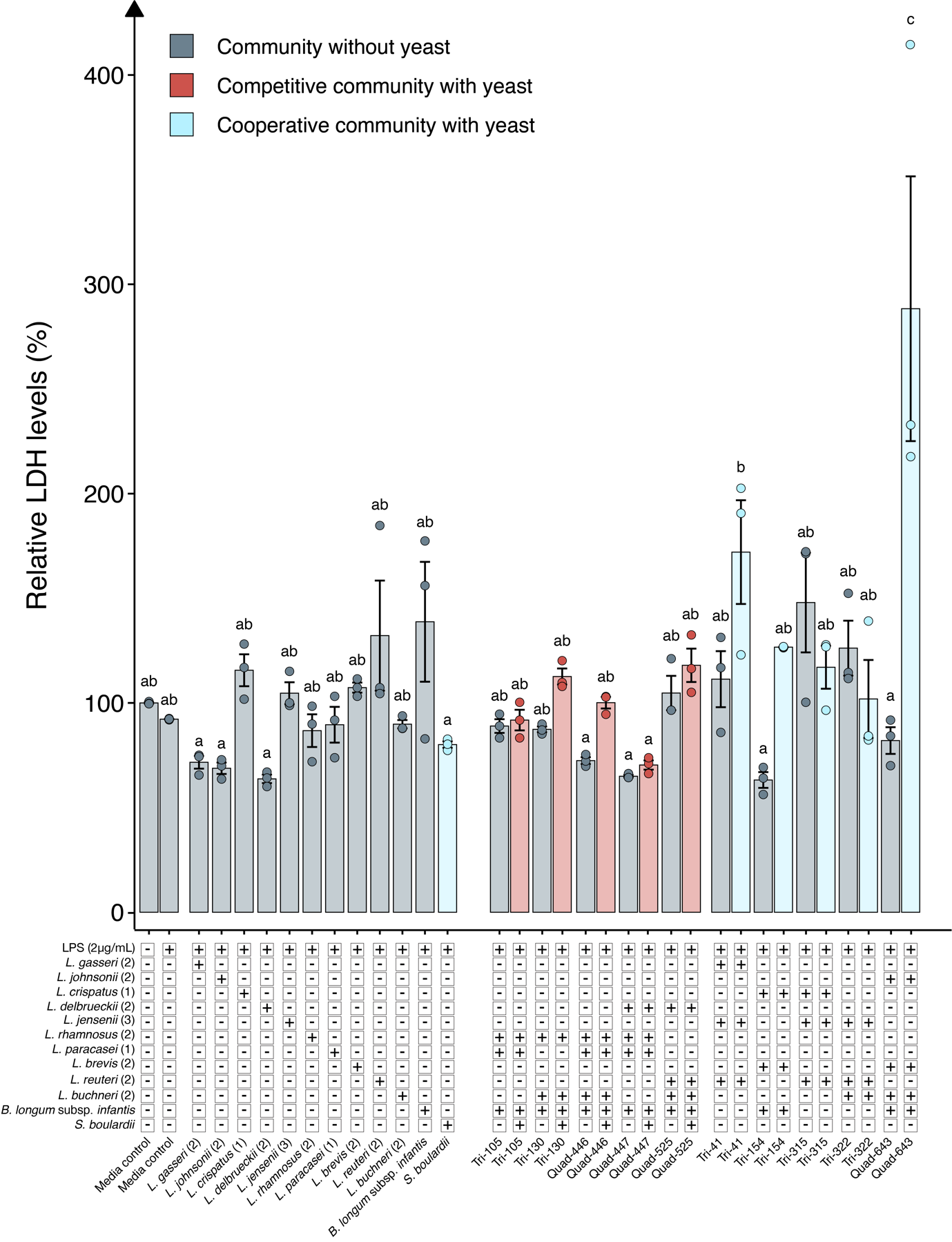
**

**Figure S19. Cellular cytotoxicity of spent media treated mammalian cells.** Relative lactate dehydrogenase (LDH) release in HT-29 cell line challenged with LPS (2 µg/mL) and spent media (10% v/v) for 24h. Data is normalised to media control without LPS. *P* values were computed using One-way ANOVA with Tukey HSD adjustment for multiple comparisons. Each letter (a, b, and c) above the bars indicates statistically distinct group. Bars labelled with the same letter indicate no significant difference between those groups, while bars labelled with different letters indicate significant differences. Significance level at *P* < 0.05.
